# Comparison of Predicted X-Ray Fiber Diffraction Patterns from All-Atom and Coarse-Grained Actin Filament Models Under Nonuniform Strain

**DOI:** 10.3390/ijms27010280

**Published:** 2025-12-26

**Authors:** Momcilo Prodanovic, Andjela Kafedziski, Thomas C. Irving, Srboljub M. Mijailovich

**Affiliations:** 1 FilamenTech Inc., Newton, MA 02458, USA; momcilo.prodanovic@kg.ac.rs (M.P.); grujic.angel@gmail.com (A.K.); 2 Institute for Information Technologies, University of Kragujevac, 34000 Kragujevac, Serbia; 3 Faculty of Physics, University of Belgrade, 11000 Belgrade, Serbia; 4 Biology Department, Illinois Institute of Technology, Chicago, IL 60616, USA

**Keywords:** X-ray fiber diffraction pattern predictions, actin filaments, all-atom simulations, coarse-grained simulations, spatially explicit model, nonuniformly strained helical structures, MUSICO

## Abstract

Small-angle X-ray fiber diffraction has informed much of what we know regarding the molecular events during muscle contraction but robust tools for predicting X-ray fiber patterns from muscle have been lacking. A complication in formulating such tools is the dynamic, stochastic nature of the sarcomere structures during contraction where individual myofilaments undergo deformations due to nonuniform strain generated by the myosin crossbridges. Here, we address this need with a “forward problem” approach using a spatially explicit model (MUSICO) to predict the molecular configurations responsible for the observed muscle force and use these configurations to predict the diffraction patterns that can be compared to experiments. We combine this with a newly developed, rigorous formulation, presented here, for the calculation of 2D diffraction patterns from actin filaments under nonuniform strain. We compare all-atom predictions to coarse-grained simulations to show how much information is lost by coarse-graining, and discuss the results in the context of diffraction patterns currently obtainable experimentally. We show that most low-resolution coarse-grained models in the literature suffice for prediction of meridional peak shapes for the purposes of estimating force distributions in the actin filaments, but accurate prediction of layer line intensities require much higher resolution models, including the all-atom models as presented here. These developments represent an important step towards our long-term goal of using molecular simulations to interpret X-ray fiber diffraction patterns from striated muscle during active contraction.

## 1. Introduction

Almost all filamentous structures within eukaryotic cells and connective tissues have helical arrangements of subunit proteins. Over the years, many experimental techniques have been developed directed at understanding the cellular processes involving fibrous molecular systems and how these processes are modulated in disease. A particularly powerful technique for studying fibrous molecular assemblies is small-angle X-ray fiber diffraction, with the most common application in biology being the study of striated muscle. The geometrically repetitive arrangements of helically arranged actin and myosin molecules in the myofibrils represent a large-scale, semi-crystalline structure that gives rise to rich X-ray diffraction patterns. Another advantage of X-ray diffraction for the striated muscle system, as opposed to, e.g., electron microscopy, is that structural information can be obtained simultaneously with the physiological data in living tissues during active contraction, providing unique opportunities to relate structure to function. X-ray diffraction, therefore, has informed much of what we know about the sequence of molecular events involved in muscle contraction [1,2,3]. It also provided early evidence for the steric blocking model of thin filament regulation [4,5,6]. More recently, it has been appreciated that thick filaments are also regulated by transitions between quasi-helically ordered, inactive OFF states of myosin and the disordered ON states of myosin available to interact with actin by strain-dependent [7,8,9,10,11] and calcium-dependent [12,13] mechanisms. The evidence for all these mechanisms, however, is based on only a small subset of all information potentially available in the X-ray diffraction patterns [13,14].

Historically, most X-ray fiber diffraction patterns collected from striated muscle have been of very low resolution, seldom extending beyond the 5.1 nm actin reflection (7th actin layer line). Recent advancements in synchrotron X-ray diffraction technology have significantly enhanced the ability to capture higher-order reflections, which have been shown to be essential for interpreting nonuniform deformation of actin and myosin filaments in contracting muscle fibers [15,16]. More detailed patterns, coupled with access to high-resolution structural information from crystallography [17,18] and cryo-electron microscopy [19,20,21,22] for many sarcomere components, provide an opportunity to detect and interpret finer movements of the sub-molecular structures within contracting muscle fibers. To realize this potential, the development of more advanced computational tools is urgently needed to interpret both existing and future X-ray diffraction data and address many unresolved controversies surrounding muscle contraction.

Straightforward application of crystallographic approaches to the muscle system are unlikely to be fruitful because of the dynamic nature of the sarcomere during contracting, where, rather than having a single structure, there is an ensemble of structures. Individual filaments are also not static but undergo deformations due to nonuniform strain along the filament lengths. Such complex systems are most likely to be amenable to a “forward problem” approach where a multiscale model incorporating these complexities is constructed and used to predict the diffraction patterns that can be compared to experiments and iteratively refined. Atomistic modeling approaches applied to large sarcomere structures would potentially be the most scientifically valuable, but they are computationally expensive. Moreover, it is not known what level of resolution in the model is needed to be sufficient to accurately simulate the existing and future X-ray fiber diffraction pattern observations and what additional insights could be gained by obtaining experimental data to higher resolution.

Interpretations of X-ray fiber diffraction patterns from filamentous biological structures are usually based on the Fourier transform of helices with fixed periodicities [23,24,25,26]. Direct application of classical helical diffraction theory to reconstruct the movements of proteins in living cells is hindered by two main challenges: (i) the inability to decompose the integral effects of multiple reflections from various helical structures, and (ii) the effects of distortions in these helical structures caused by intracellular forces. These limitations restrict our ability to fully extract the information potentially available in X-ray diffraction patterns. This motivated the development of a new theoretical approach for calculating fiber diffraction patterns from nonuniformly distorted helical molecules [15]. The nonuniformity of monomer spacings in the thin filaments in contracting muscle arises from forces transferred from the myosin filaments to actin filaments through the attachment of crossbridges. Using this methodology, we calculated the distributions of spacings caused by crossbridge action along actin filaments in contracting muscle, employing spatially explicit simulations of muscle contraction [27]. These simulated data enabled us to reproduce realistic intensity profiles and spacing values for deformed actin filaments [16], closely resembling the observed meridional profiles in data from H.E. Huxley and K. Wakabayashi [28,29]. Furthermore, these simulations allowed us to map the distribution of forces within individual actin filaments, demonstrating the potential to extract significantly more information about molecular structure and function from X-ray fiber diffraction data, specifically, from contracting muscle, and potentially from any coherently diffracting helical structure in living cells.

In our earlier work [15,16], the complex, helically arranged monomer structure was represented as a single discontinuous helix, with subunits arranged along a continuous wire exhibiting piecewise change in helix pitch and an equivalent helix radius. In this simplified approach, each subunit is represented as a single sphere with an adopted equivalent radius and mass [15,16,30]. This approach was sufficient to predict the changes in meridional X-ray diffraction patterns from contracting muscle during mechanical transients. However, this approach restricts the analysis of X-ray diffraction patterns to meridional reflections, as the predicted off-meridional reflections are of limited value, due to the spatial distributions of atoms in radial, axial, and azimuthal directions within each monomer.

Previous attempts to simulate the diffraction patterns from muscle used coarse-grained approaches to represent the atomic-level molecular substructures. The study of Koubassova et al. [31], for example, was able to obtain reasonable fits to the fiber diffraction pattern from muscle in rigor out to the 5.1 nm actin layer line using a model with 1 nm-radius spheres so that the actin monomers were represented by nine spheres, providing resolution up to 12 nm. A more detailed model, consisting of 47 spheres each with a 0.6 nm radius, provided 4 nm resolution. This raises the question as to how much better could be performed using higher resolution approaches.

The overall goals in this study were to (1) develop a rigorous methodology to calculate the predicted 2D fiber diffraction pattern from actin filaments under nonuniform strain (2) using the all-atom calculations as a standard, rigorously assess the amount of information lost in the coarse-grained approach and how this loss could affect interpretation, and (3) determine what would be the simplest coarse-grained model adequate to use simulations of peak widths to estimate nonuniform strain distributions. We compared the X-ray diffraction patterns generated by different coarse-grained models against those computed from the all-atom actin monomer structure, allowing us to estimate the extent of information loss associated with different levels of coarse-graining and select an optimal coarse-grained model that balances computational efficiency with the preservation of critical structural features necessary for accurate diffraction pattern simulation. We were able to show that the changes in intensity profile shape of the actin meridional reflections due to nonuniform strain could be predicted with any coarse-grained model, including the original one sphere per actin monomer model, validating our earlier work. The results are discussed in the context of currently available diffraction patterns of muscle and how they may motivate higher resolution, more insightful experiments.

## 2. Computational Models of X-Ray Diffraction Patterns from Deformed Actin Filaments in Contracting Muscle

The general methodology for simulating X-ray diffraction patterns of deformed continuous helices and discontinuous helices was defined by Prodanovic et al. [15]. This approach was later used by Mijailovich et al. [16] to assess force distributions in the actin filaments in contracting bullfrog sartorius muscles from X-ray diffraction data. In the current study, we apply the same general methodology. However, instead of considering a single discontinuous helix passing through a representative point in each monomer along the actin filament, we consider multiple discontinuous helices, each helix corresponding to a specific atom in the first monomer and passing through the same atom in all other monomers along the filament to generate an all-atom simulation. For clarity, we have included only the essential equations from previous publications [15,16] and provided a detailed explanation of all features in the new formulation. For comparison with these all-atom simulations, we have also developed coarse-grained models with the goal of identifying models that can recapitulate most of the key features of the all-atom model but can be executed much faster and with more moderate computational resources.

### 2.1. Origins of Nonuniform Deformation of the Actin Filament Helices

**Figure 1:**
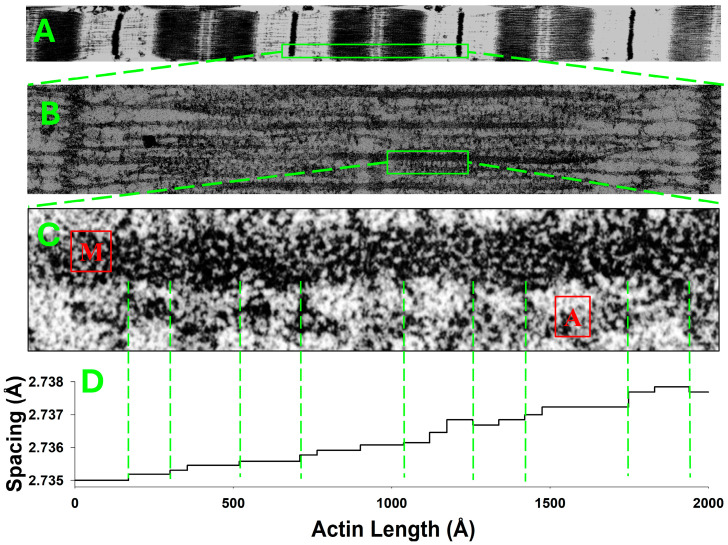
The monomer spacings along an actin filament under nonuniform strain during fully developed isometric tension. (**A**) Electron micrograph (H.E. Huxley unpublished) showing part of myofibril, and (**B**) zoomed in detail from panel (**A**) showing interdigitated actin (denoted by a red A in panel (**C**)) and myosin filaments (denoted by a red M in panel (**C**)) in frog (skeletal) sarcomere lattice. (**C**) Zoomed in detail from (**B**), showing the position of myosin heads attached to actin filaments (crossbridges), denoted by green dashed lines. (**D**) The piecewise change in spacing between neighboring actin monomers coincides with the locations of myosin molecules bound to actin sites. Here interactions are shown between only one myosin filament with one actin filament (green dashed lines between panels (**C**) and (**D**)), but in the hexagonal lattice in the A-band, three myosin filaments will interact with each actin filament. Actin monomer spacing generally increases from the free end of the actin filament toward the Z-line but can occasionally decrease due to the effect of stochastic compressive forces by the bound crossbridges.

In relaxed actin filaments, the axial inter-subunit spacings are nearly uniform, with minor variations typically caused by thermal fluctuations. However, in living cells, each filament interacts with other filaments or intracellular structures, causing deformation of the filament. The most extensively studied structures of this kind are in contracting striated muscle fibers, which are composed of axially connected subunits called sarcomeres (Figure 1A).

The sarcomere lattice contains interdigitated actin and myosin filaments (Figure 1B), where myosin heads interact with binding sites on the actin filaments. These interactions are driven by the actomyosin cycle and regulated by various regulatory proteins. The cycle includes the formation and disruption of actin–myosin connections, called crossbridges, at different locations along the interacting filaments (Figure 1C). Bound myosins, in their various actomyosin states, transfer variable amounts of force between myosin and actin filaments. The force along each filament changes at the positions of crossbridge attachments. The cumulative force typically increases, and occasionally decreases, from zero at the filament tips towards the M-line in myosin filaments and towards the Z lines in actin filaments.

In contracting muscle, actin filaments primarily bear axial forces, resulting in axial deformation along the filaments, except in the region near crossbridge attachments to actin. In this region, force is transferred over an interaction area, causing complex deformations in the proximal actin monomer structure. For simplicity, we neglect these local deformations near the bound crossbridges and assume that the force exerted by each bound myosin crossbridge is transferred at a single axial position along the actin filament. This position is defined as a plane passing through the center of the monomer mass, perpendicular to the thin filament axis. This assumption will impose very small errors in the predicted X-ray patterns because the effects of force transfer are accurately diffracted from all other actin filament regions, except from minor local distributed deformations near the binding site. Therefore, without loss of generality, we assume that the force along actin filaments changes in a piecewise manner, with the step-change in force between segments equal to the crossbridge force transferred. Consequently, these piecewise force distributions are reflected in the corresponding piecewise monomer spacing, as illustrated in Figure 1D, where the monomer spacing is derived from explicit 3D model simulations of contracted muscle. Due to the discrete, stochastic attachments of myosin heads to actin sites on actin filaments in the 3D sarcomere lattice (Figure 1C), the stepwise change in strain (Figure 1D) follows the changes in force along the filaments.

### 2.2. Calculation of Nonuniform Deformation Along Actin Filaments in Contracting Muscle

The development of the computational platform MUSICO (MUscle SImulation COde) [16,27,32,33,34] has provided a valuable tool for studying X-ray diffraction patterns in muscle. It enables simulations of nonuniform filament deformation in contracting striated muscle fibers using Monte Carlo calculations within a spatially explicit 3D sarcomere lattice. MUSICO was originally created to model only muscle mechanical responses, with its mature form first reported in 2016 [27]. Since then, it has been expanded to simulate X-ray diffraction patterns using 3D multiscale models [15,16].

**Figure 2:**
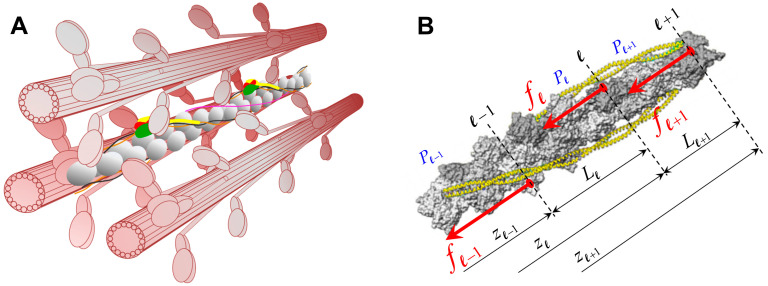
Spatially explicit simulations of muscle force using MUSICO. Myosin filament interacts with actin filaments, arranged in multiple interconnected hexagonal lattices. (**A**) Three-dimensional representation of three myosin filaments interacting with an actin filament. (**B**) The crossbridge forces in the axial direction calculated in MUSICO (red arrows) are shown on the surface 3D structural model of actin filament. Note that positions of axial forces follow helical position of actin binding sites where myosin heads are bound. The subscript l denotes the current number of the plane where myosin is bound to actin and crossbridge force acting on actin is $f_{l}$. The length of segments, $L_{l}$, are defined by the axial positions of the planes, and the pitch within the segment is $P_{l}$.

The actin–myosin interactions within the sarcomere lattice are specified in the context of the discrete lattice structure formed by the interdigitated actin and myosin filaments. In vertebrate striated muscle, myosin and actin filaments are organized into a regular hexagonal lattice, with three myosin filaments surrounding each actin filament (Figure 2A), and six actin filaments around each myosin filament [35].

For prescribed boundary conditions and levels of activation, MUSICO simulations provide the coordinates of each monomer along the filaments in deformed configuration. Thus, at any instant of time, the force transfer from myosin crossbridge to actin (Figure 2B) deforms the actin filaments along the filament length, forming a piecewise distribution of forces, strains, and therefore, the intermonomer spacings. The location of myosin bound to the actin filament, based on the calculated monomer coordinates in a deformed configuration, is defined as the distance between the free end of the actin filament, $z_{0}=0$, and the midplane through the interaction area where a crossbridge is bound, $z_{l}$. This midplane corresponds to the plane passing through the center of mass of the actin monomer, where l denotes the plane number (Figure 2B). Because the tension in the actin filament changes at each site of bound myosin, the intermonomer spacings will also change. Thus, the forces accumulate from the free end of actin filament toward the Z-disk, and the changes in intermonomer spacings follow the same pattern (Figure 1D).

### 2.3. The Fourier Transform of Multiple Discontinuous Helices with Finite-Size Atoms or Coarse-Grains

**Figure 3:**
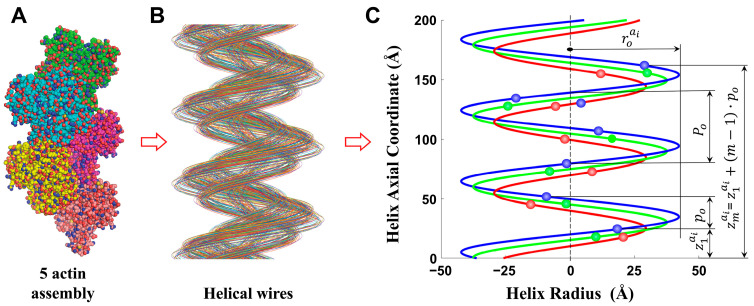
All-atom representation of actin helices. A fragment of an actin filament consisting of five helically arranged subunits (from 3actin.pdb structure [36]) is shown in (**A**). Through each atom, $a_{i}$, of a subunit m=1, one helical wire can be constructed to pass through the same atom in all other subunits. For each of the 2991 atoms, there will correspond one helical wire with its own helix radius, $r^{a_{i}}$, and pitch, P (**B**). The discontinuous helix of an array of atoms, $a_{i}$, repeating along all monomers with axial spacing, p, can be constructed for each helical wire (**C**), where the coordinates of the atom in monomer m are $r_{m}^{a_{i}}$, $\psi_{m}^{a_{i}}$, and $z_{m}^{a_{i}}$. The expression for $z_{m}^{a_{i}}$ of an undeformed helix is shown as a function of monomer spacing and pitch, $p_{o}$ and $P_{o}$.

Predicting the diffraction pattern from an actin filament requires accounting for the helical arrangement of its subunits and the fact that each atom (or coarse-grained sphere) follows a unique helical path along the filament. In our formulation, each atom defines a separate “discontinuous helix”: a helical trajectory that is sampled only at the axial positions of the monomers. The total diffraction pattern of the filament is obtained by summing the contributions from all such helices (Figure 3).

In relaxed filaments, these helices are nearly periodic, producing sharp and well-defined meridional and off-meridional reflections. During contraction, however, crossbridge forces introduce nonuniform, piecewise changes in monomer spacing, which distort the helices and broaden or shift specific reflections. Our approach extends earlier single-helix formulations [15,16] by treating the filament as an assembly of thousands of parallel discontinuous helices, each with its own radius, azimuthal phase, and finite spatial extent defined by the atomic coordinates.

For coarse-grained models, groups of atoms are replaced by equivalent spherical grains, each of which defines its own helical trajectory. This reduces computational cost but also limits resolution due to the grain size and the reduced number of available helical radii. Our goal here was to critically characterize these limitations in the context of predicted diffraction patterns, as shown in the Results Section below.

All mathematical expressions, definitions of geometric parameters, and the complete derivation of the Fourier transform for multiple discontinuous helices are provided in Appendix A, Appendix B, Appendix C, Appendix D, Appendix E and Appendix F. A comprehensive list of all symbols and parameters is given in Appendix G, Table A1.

## 3. Results

### 3.1. X-Ray Diffraction Patterns Predicted from All-Atom Structural Model of Actin Filaments

**Figure A1:**
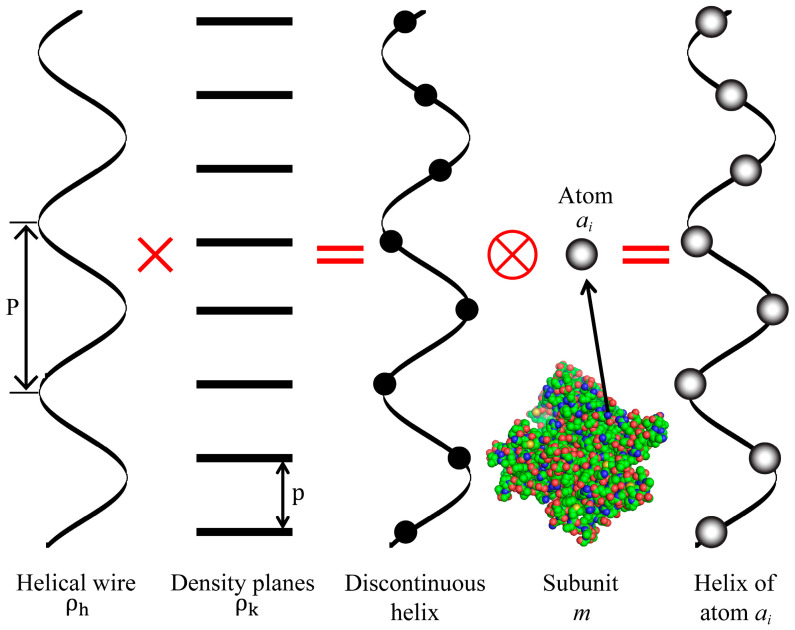
Basic principles of constructing a discontinuous helix. The convolution of a single turn of the helix and linearly arranged 3D delta functions (lattice of points) aligned along the helix axis and separated by the pitch, P, forms helical wires, $\rho_{h}$, of prescribed length [30]. For helically arranged subunits or a multi-atom structure, the axial position of each monomer or atom can be represented by its own density plane, $\rho_{k}$, axially separated by distance p. Then, the discontinuous helix can be formed by product of the helical wire and $\rho_{k}$ for dimensionless and massless subunit or atom. For helically arranged subunits m consisting of many atoms, each atom, $a_{i}$, forms its own wire and discrete helix. The helical arrangement of an atom $a_{i}$ in actin monomers along an actin filament can be represented as convolution of discontinuous set of the atom positions and the atom object function represented as sphere with its Van der Waals radius.

Ideal X-ray fiber diffraction patterns can be calculated from all-atom representations of actin filaments in both resting and deformed configuration by considering the radial and axial position of each atom in the actin monomers. The positions of specified atom, $a_{i}$, in each monomer along the actin filament form a discontinuous helix, and actin filament is represented by the large number of discontinuous helices (Figure 3). The helices are shifted azimuthally depending on spatial position of atom within a monomer, and it is usually defined by the atom position in the first monomer under relaxed conditions (for details see the Methods Section). Furthermore, the discontinuous helix consists of finite atomic-sized subunits (Figure A1), determined from the Van der Waals volumes and electron densities of atoms in the actin monomer structure. All atoms of the actin monomer, except hydrogen, are included in the pattern calculations, as hydrogen atoms scatter weakly and contribute negligibly to the predicted X-ray diffraction pattern. The predicted patterns can serve as reference standards for evaluating simplified approaches based on coarse-grained molecular structures, which may often be adequate for analyzing diffraction patterns obtained with current X-ray technologies.

**Figure 4:**
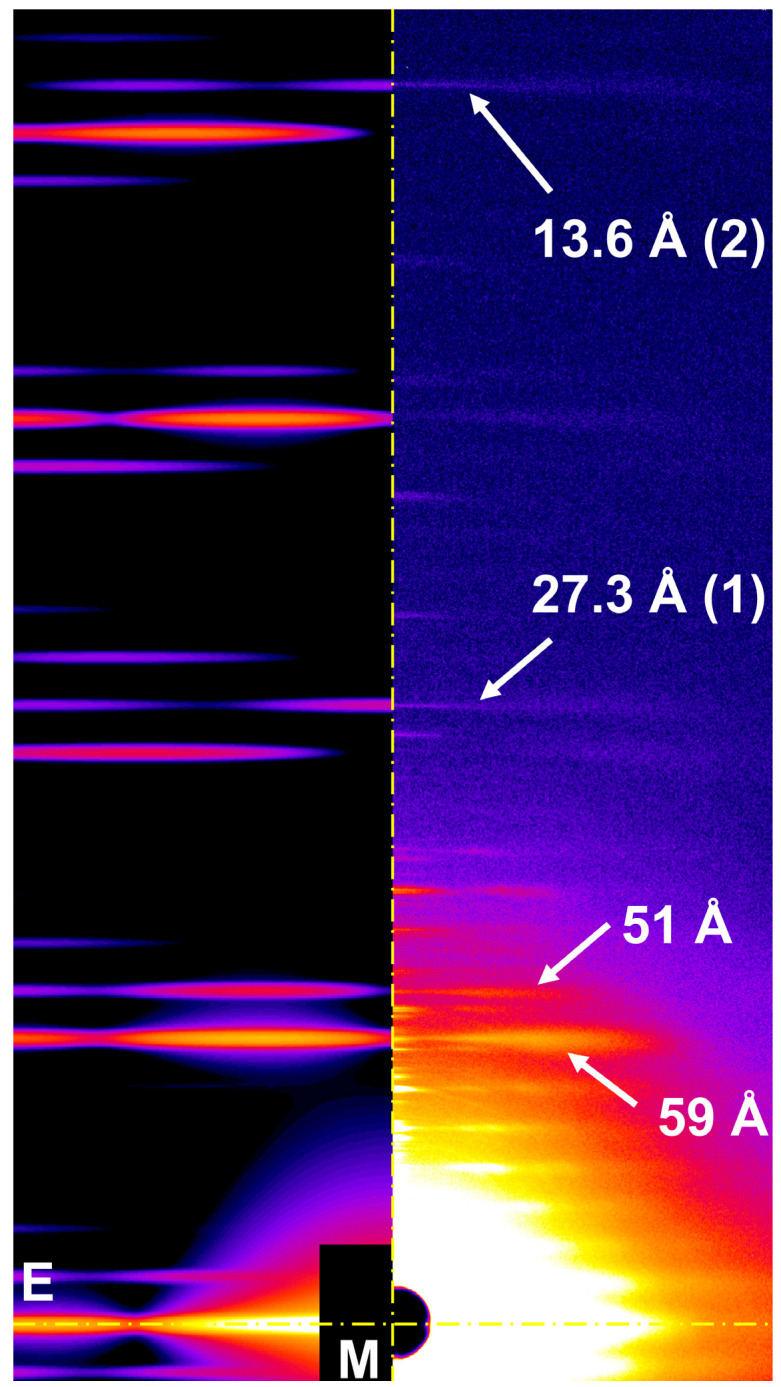
Comparison of predicted and X-ray diffraction patterns for a relaxed actin filament (all-atom model) with an observed X-ray diffraction pattern from relaxed frog muscle adapted from [37]. Arrows indicate the positions of the first two actin meridional reflections and the 51 Å and 59 Å layer lines.

Figure 4 compares an all-atom predicted X-ray diffraction pattern from relaxed actin filaments with an observed diffraction pattern from frog muscle [37]. Our predicted actin meridional reflections quantitatively matched positions of the observed layer lines at 59 Å and 51 Å, first actin meridional reflection (at ~27.3 Å) and the second (at ~13.6 Å), as well as near meridional layer lines. There is much more detail in the predicted pattern than in the experimentally observed patterns, however, indicating that actin filaments in muscle are disordered, which will need to be considered in quantitative comparisons of prediction and experiment. The overall qualitative agreement in positions and magnitude of the reflections with experimental data encouraged us to further explore the diffraction patterns of both relaxed and contracted muscle using an all-atom computational model.

**Figure 5:**
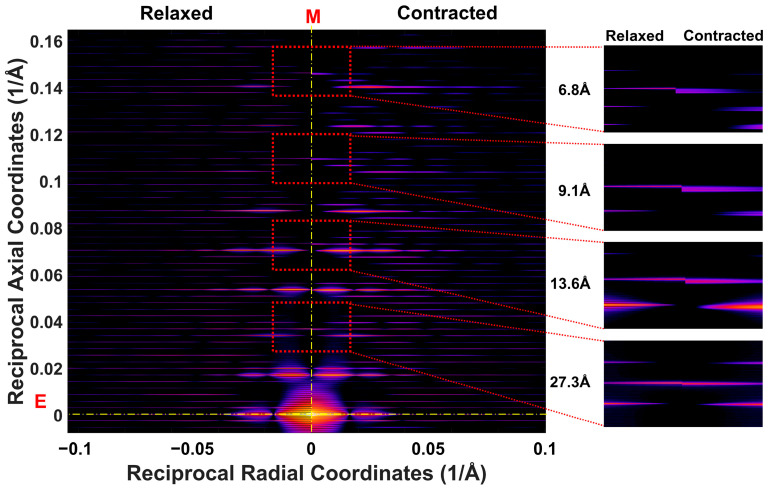
Predicted X-ray diffraction patterns of relaxed and contracted actin filaments (all-atom model). Diffraction intensities computed from an all-atom model of the actin filament, sampled over 18 viewing angles, are presented for both relaxed (**left**) and contracted (**right**) states, providing insights into the mechanical behavior of actin under nonuniform strain. Red letters, E and M, and yellow dash-dotted lines, denote equatorial and meridional lines respectively. The insets display detailed profiles of the meridional reflections at: 27.3 Å (1st actin meridional reflection), 13.6 Å (2nd), 9.1 Å (3rd), and 6.8 Å (4th), along with nearby layer lines. These profiles illustrate systematic shifts in reflection spacings and broadening of meridional peaks when filaments are strained during contraction.

**Figure 6:**
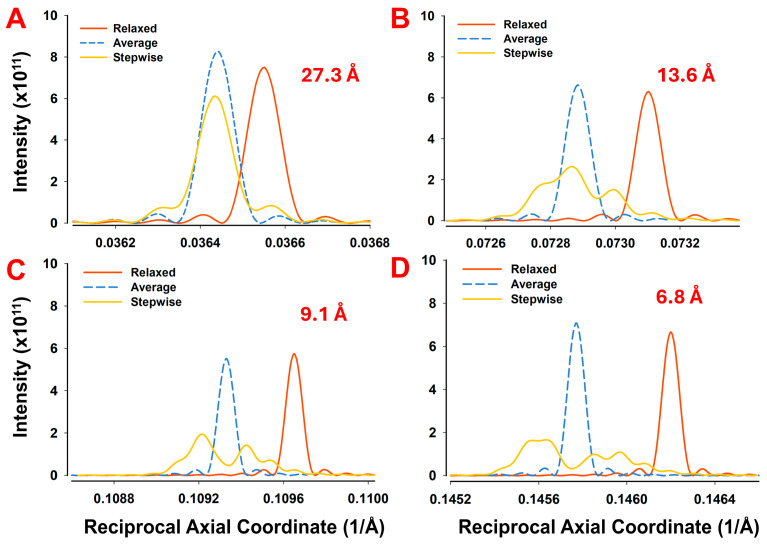
Comparison of meridional actin filament profiles between relaxed and contracted muscle from Figure 5 (all-atom model) for the first (**A**), second (**B**), third (**C**), and fourth actin meridional reflections (**D**). Meridional intensity profiles predicted by MUSICO simulations are compared for three scenarios: (i) relaxed filaments (red thick lines); (ii) uniformly deformed filaments exhibiting a constant Mean monomer spacing (blue dashed lines); and (iii) filaments with nonuniform monomer spacings resulting from the local change in force along actin filament due to crossbridge forces from bound myosins (orange thick lines) (see Figure 1B). The relaxed and uniformly deformed filaments show similar profiles with reciprocal space shifts corresponding to changes in spacing. In contrast, the meridional reflections from nonuniformly deformed filaments exhibit broader diffraction distributions reflecting the degree of nonuniformity, except for the 1st actin meridional reflection at ~27.3 Å.

Predicted X-ray diffraction patterns from actin filaments in relaxed and contracting muscle, obtained through all-atom simulations, are visible up to the 12th-order actin meridional reflection, corresponding to a resolution of 2.17 Å, Figure 5 shows the predicted full 2D diffraction pattern up to the fourth-order meridional reflection to qualitatively illustrate the level of detail in these patterns and how nonuniform strain in the filaments shifts and axially broadens the meridional peaks and layer lines in the contracting patterns. These effects can be seen more quantitatively in the axial intensity profiles of the first four meridional reflections, as shown in (Figure 6). The profiles of the first-order meridional reflection (Figure 6A) are relatively insensitive to nonuniform strain, allowing estimation of the average axial actin monomer spacing in resting and contracting muscle, which can be related to filament stiffness, as shown previously by H.E. Huxley et al. [28] and K. Wakabayashi [29]. In our simulations, both uniformly and nonuniformly deformed filaments experience an average axial strain of ~0.3% relative to the relaxed state. The intensity of the first actin meridional reflection is ~10% higher in uniformly deformed than in the relaxed filaments, whereas the peak height of the nonuniformly deformed filament is reduced by ~19% relative to relaxed.

In contrast, the effects of nonuniform strain, resulting from the cumulative effects of attached force-producing crossbridges from the tip of the actin filament to the Z-line, are evident in the axial profiles of higher-order meridional reflections of (Figure 6B–D). These profiles become progressively broader by approximately 4-, 6-, and 8-fold relative to relaxed filaments, and increasingly distorted, with the intensities falling sharply in nonuniformly deformed filaments by ~60% (2nd), ~66% (3rd), and ~75% (4th), reflecting the effects of nonuniformity in spacings along the filaments [16]. These results from all-atom simulations show a similar shape and position of the reflections to those previously calculated using a simplified approach involving a single helix joining all monomers along the actin filament [15,16], where the model predictions were compared with the experiments up to the second-order actin meridional reflection.

### 3.2. X-Ray Diffraction Patterns Predicted from Coarse-Grained Structural Model of Actin Filaments

The coarse-grained models represent the actin monomer by varying number of grains, modeled as equivalent spheres whose volumes approximate the local electron density distribution of approximately equal-sized fragments of the actin monomer. Similarly to the all-atom model, each grain is characterized by the radius of its helical cylinder, $r_{o}^{cg_{i}}$, the size of its the equivalent sphere, $r_{s}^{cg_{i}}$, and the spatial position of specified grain within the first monomer of F-actin (See Figure S1).

The principal effects of increasing the number of grains per actin monomer are (i) an increased number of radial positions of grain centers, approaching those of the all-atom system; and (ii) a reduction in grain size, reducing the influence of the grain’s shape factor on the predicted intensities that can limit maximum resolution of observable predicted reflections, as well as artefactually reduce their intensities. The angular and axial shifts in the discontinuous helices, which depend on the position of each grain in the first monomer have only minor effects. The effect of the angular shifts is further minimized by cylindrical averaging of the diffracted pattern intensity.

**Figure 7:**
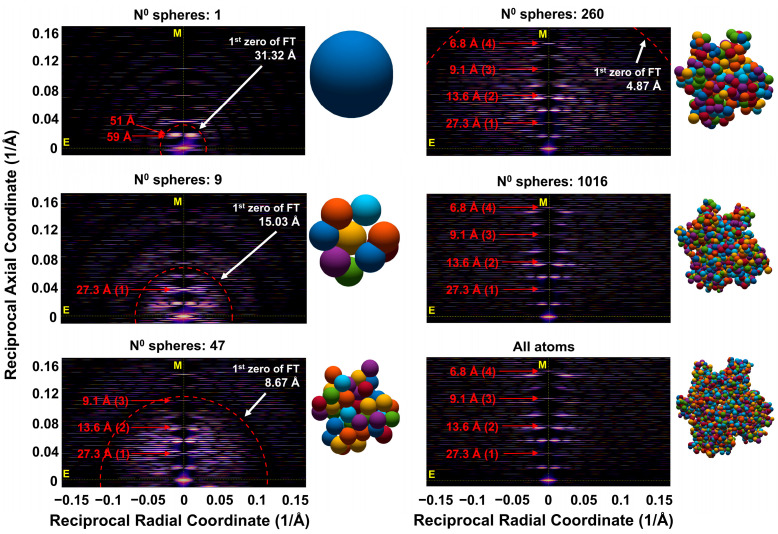
Comparison of predicted all-atom and coarse-graining models of relaxed actin filaments, at Ψ=0. As the number of grains decreases, the size of grains (equivalent sphere radius) increases, leading to a reduction in the visible field. The outer boundary of the visible field is indicated by dashed red line semi-circles, which show the first zero of Fourier transform of the equivalent sphere, as given in Table 1.

The combined effects of these factors are summarized in Figure 7, which compares the predicted X-ray diffraction patterns obtained from simulations of F-actin X-ray diffraction patterns with varying degrees of coarse-graining, specifically models containing 1, 9, 47, 260, 1016 grains and all-atom representation per actin monomer. As the equivalent grain size increases, defined by the radius of equivalent sphere, $r_{s}$, the number of visible layer lines decreases. This limits the radial extent of observable diffraction features. The intensity distributions in the patterns are modulated by the intensity transform of the spherical grains, i.e., the form factor for a spherical object. The radius corresponding to the first zero of these transforms, given in Table 1, is marked by a red dotted circle in Figure 7 and corresponds to circular regions of reduced intensity in the diffraction images. In all-atom simulations, the predicted X-ray diffraction patterns display features extending well beyond the fourth actin meridional reflection. However, as the grain size increases, e.g., for example to $r_{s}^{cg_{i}}=6.2$ Å, corresponding to 47 spheres per monomer, only the first three actin meridional reflections remain visible. With further increases in grain size, such as to $r_{s}^{cg_{i}}=10.75$ Å in the case of nine grains per monomer, only the first actin meridional reflection is still visible. In addition to limiting the radial extent of observable reflections, the strong curvature of the intensity transform of the spherical grains implies that the intensities of visible reflections inside the limiting circle, denoted by dashed lines in Figure 7, become increasingly attenuated as you approach the zero values for the transform (Table 1).

### 3.3. Sensitivity Analysis of Predicted X-Ray Diffraction Patterns on Parameters of Structural Model of Actin Filaments

To better understand the sources of limitations in X-ray diffraction predictions from coarse-grained representations of the F-actin monomers, we performed a sensitivity analysis on the reduced number of grains by quantifying the effects of (i) reduced number of $r_{o}^{cg_{i}}$ representing radial position of the centers of the grains, (ii) increased average size of grains, $r_{s}$, and (iii) distributions of grain sizes, $r_{s}^{cg_{i}}$, as a function of the degree of coarse-graining. The predicted X-ray diffraction patterns are strongly affected by the discontinuous helix cylinder radius, $r_{o}$, and the grain size represented as an equivalent sphere radius, $r_{s}$, which, in the limiting case, reduces to atomic dimensions. To understand their individual contributions, we performed a sensitivity analysis focusing on effects of the helix cylinder radius, $r_{o}$, passing through the center of an atom or a coarse-grained structure, as well as the effect of atomic size or equivalent grain size on the predicted X-ray diffraction pattern.

#### 3.3.1. The Effects of Helix Cylinder Radius on the Predicted X-Ray Diffraction Pattern

**Figure 8:**
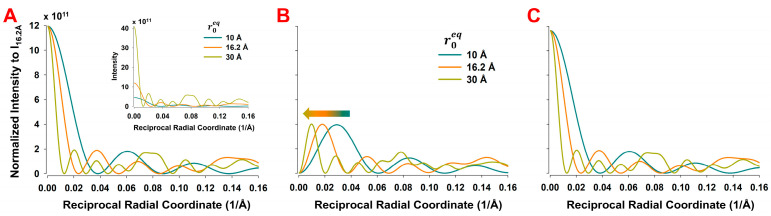
Sensitivity analysis of profiles along layer lines for discrete helix cylinder radii in relaxed actin filaments. All plots are normalized to the intensity of a helix with radius of 16.24 Å. (**A**) Normalized equatorial profiles. The inset shows the same profiles before normalization, illustrating the effect of the helix radius on their magnitude. (**B**) Normalized radial profiles at the level of 6th actin layer line (59 Å). The arrow shows the inward shift of the first maximum with decreasing helix radius radius (**C**) Normalized radial profiles at the level of the 1st actin meridional reflection.

While the effect of the shape factor of the coarse-grains is easy to understand, a more subtle effect is on the radius of helical paths joining the centers of the coarse-grains. The helix radius, $r_{o}$, affects the magnitude of reflections and the shape of the Bessel functions. In Figure 8, we compare three different helix radii: the radius corresponding to the center of mass of the actin monomer, $r_{o}$ = 16.2 Å, a smaller radius of 10 Å, and a larger radius of 30 Å. The differences in 2D X-ray diffraction patterns for these three radii are subtle but become more apparent when examining the differential intensity maps between the predicted patterns at $r_{o}$ = 16.2 Å vs. $r_{o}$ = 10 Å and at $r_{o}$ = 16.2 Å vs. $r_{o}$ = 30 Å (see Figure S2). These difference maps demonstrate that variations in the helix cylinder radius significantly affect the intensity magnitude more significantly along the R-coordinate in predicted 2D X-ray diffraction images. The quantitative differences are illustrated as intensities of radial profiles along the layer lines (Figure 8). Notably, the intensities significantly increased with an increase in $r_{o}$, as shown in inset of Figure 8A.

The magnitude of the Fourier transform, $\hat{f}\left(R,\Psi ,Z\right)$, depends on $r_{o}$ (see Equation (A4)) and the intensity on $r_{o}^{2}$ (Equation (A7)). Thus, for comparisons of the shapes of the profiles along layer lines, we have normalized the magnitude of the Bessel functions by dividing them by a factor $r_{o}^{2}$, at $r_{o}$ = 16.2 Å. We compared the predicted normalized profiles for $r_{o}$ = 10 Å, 16.2 Å, and 30 Å. Figure 8A shows a comparison of the equatorial profiles, Figure 8B shows radial profiles along the 6th actin layer line (59 Å), and Figure 8C displays radial profiles across the first meridional actin reflection (27.3 Å). Of particular importance is the behavior of the off-meridional peaks (Figure 8B), which become narrower and progressively shift toward the meridional axis as the helix radius increases. Taken together, layer line profiles predicted by coarse-grained models can deviate significantly from those of all-atom models, depending on the number and spatial distribution of the helix radii imposed by the size of the coarse-grains.

#### 3.3.2. The Effects of Equivalent Grain Size on the Predicted X-Ray Diffraction Pattern

**Figure 9:**
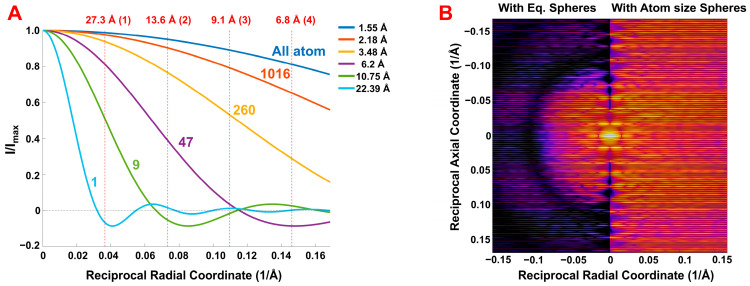
The effect of grain size on the shape factor. (**A**) Fourier transform of equivalent sphere profile for a range of average grain sizes corresponding to variety of grains per myosin monomers. Number of grains per actin monomer (blue) and average grain radius and their size associated with color of each profile line strongly affect the shape of profile. Larger gain sizes attenuate intensities of predicted X-ray diffraction patterns by rapid intensity decay at smaller reciprocal radii (Table 1); (**B**) predicted X-ray diffraction patterns for relaxed coarse-grained model with 47 spheres per actin monomer with average grain size $r_{s}$ = 6.20 Å (left side) and with average atomic size of $r_{s}$ = 1.55 Å with equivalent grain mass (right side).

The equivalent grain size, defined by radius of equivalent sphere, $r_{s}$, limits the number of visible layer lines (Figure 7), i.e., extent in the meridional direction of the X-ray diffraction pattern, and as well as the observable range in the radial direction. In all-atom simulations, the predicted X-ray diffraction patterns extend far beyond the fourth actin meridional reflection. The net effect of the grain size, representing parts of actin monomer defined in coarse-grain procedure, is defined as a “shape factor” which attenuates the intensity of predicted X-ray diffraction patterns, where the magnitude of reflection is decreased by magnitude of the sphere Fourier transform. The shape factor is calculated for each grain, and it is used to predict the pattern for each discontinuous helix passing through the specified grain center. The dependance of the shape factor on grain size can be illustrated by the degree of coarse-graining as a function of the average effective grain radius and its variation. (Figure 9).

Regarding the average effective radius, the rapid decay of intensity along the layer lines with increasing equivalent radius, $r_{s}$, is evident in the comparison of predicted 2D X-ray diffraction patterns (Figure 7 and Figure S4) of equatorial profiles (Figure S5) and meridional profiles (Figure S6). The shape factor strongly depends on the grain size, via equivalent radius, $r_{s}$, reducing magnitude of 2D X-ray reflections to very low values at reciprocal coordinate inversely proportional to $r_{s}$ (Figure 9A). Thus, for smaller grain size, as, for example, for atomic size of 1.55 Å, the shape factor reduces the magnitude of the fourth actin meridional reflection (~6.8 Å) by ~20%, whereas for 47 grains system with grain size of $r_{s}$ = 6.20 Å, the intensity is reduced to about 0% at the level of third actin meridional reflection (~9.1 Å), and significantly reduces the second actin meridional reflection at 13.6 Å (Figure 9B). Higher-order actin meridional reflections, e.g., the 3rd and 4th, require much finer resolution, with models composed of at least 260 spheres.

Reducing the size of the equivalent grain radius to atomic dimensions while preserving the total mass of the actin monomer allows us to assess how the coarse-grained helix cylinder radius affects diffraction pattern predictions relative to all-atom model simulations without the complication of shape factor of the spherical grain. As an illustrative example, we compared X-ray diffraction patterns of the coarse-grained model with 47 grains, with effective sphere size of $r_{s}$ = 6.20 Å (left half in Figure 9B), with the average atomic size sphere of $r_{s}$ = 1.55 Å (right). The use of atom size grains significantly reduces the effect of the shape factor; however, the effect of the sparce distribution of helix cylinder radii imposed by the effect size of the grains (discussed under Section 3.3.1 above) remains and can lead to significant differences in the layer line intensity distributions from all-atom predictions.

### 3.4. X-Ray Diffraction Patterns Predicted from Coarse-Grained Structural Model of Actin Filaments with Grain Size Normalized to Atomic Size Spheres

**Figure 10:**
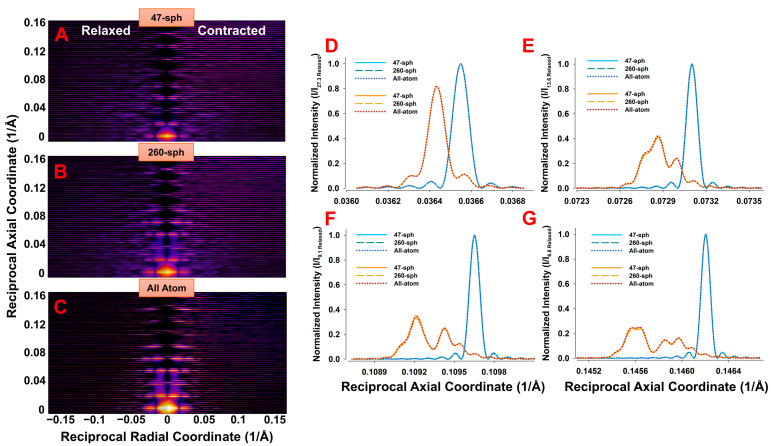
Comparison of X-ray diffraction patterns and meridional intensity profiles between relaxed and contracted actin filaments using models with different levels of coarse-graining: 47-sphere model (**A**), 260-sphere model (**B**), and all-atom model (**C**). Intensity profiles of the 1st to 4th actin meridional reflections are shown in panels (**D**–**G**), respectively. Blue lines represent relaxed filaments, while orange lines correspond to contracting filaments. The 47-sphere coarse-grained model is shown as solid lines, the 260-sphere model as dashed lines, and the all-atom model as dotted lines. The overall peak positions remain similar across different model resolutions, but the intensity varies with the level of coarse-graining. As the number of spheres in the model increases, the diffraction intensities become closer to those of the all-atom model.

The predictions of X-ray diffraction patterns from an all-atom discontinuous helix were compared with coarse-grained models of 47 and 260 spheres per actin monomer after normalization to atomic size grain size distributions (Figure 10). All panels were corrected for Lorentz effects. The resulting 2D diffraction patterns (Figure 10A–C) show that the 260-sphere model closely matched the all-atom case, while the 47-sphere model showed clear deviations, particularly in higher-order reflections. For further comparison, meridional intensity profiles were additionally normalized to the maximum of the corresponding relaxed first meridional reflection peak and presented for the 1st–4th actin meridional reflections (Figure 10D–G). With this normalization, the meridional profiles become nearly self-similar across models, with comparable peak positions and shapes. Without such normalization, significant differences in absolute intensities remain, particularly for the 47-sphere model, reflecting the effect of helical radii distributions.

To quantify these differences, the cylindrical averages of the actin meridional reflection profiles were calculated for each model and condition. The 1st reflection was nearly identical across all models, while the 2nd–4th reflections were strongly underestimated in the 47-sphere model relative to the all-atom case. The 260-sphere model remained in closer agreement. Ratios of relaxed-to-contracted peak integrated intensities were preserved across all models, with deviations below ~5% for the 47-sphere model and below ~3% for the 260-sphere model compared to the all-atom system.

**Figure 11:**
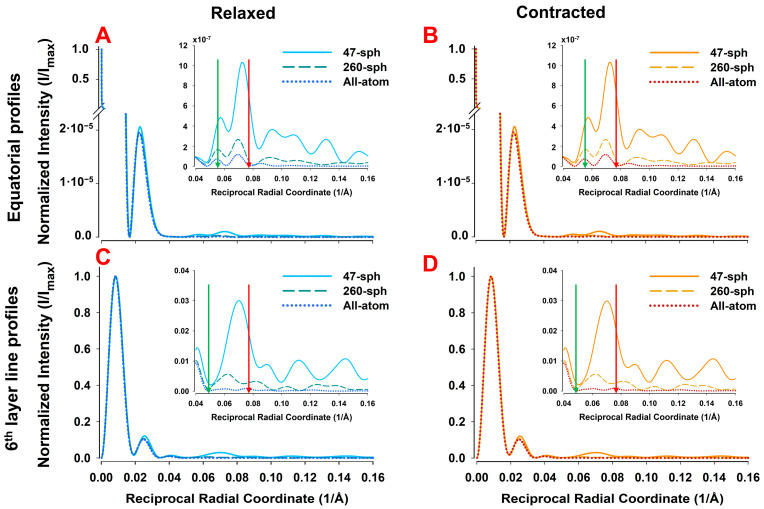
The X-ray diffraction profiles in radial direction for coarse-grained models with 47 spheres (solid lines), 260 spheres (dashed lines), and the all-atom model (dotted lines) in both relaxed (**left**) and contracted (**right**) filament states. The profiles are normalized to the intensity of the first actin meridional reflection. The insets provide a detailed view of the differences between the models, showing variations in peak positions and intensities. The red arrows indicate the extent of experimental observations if diffraction data is recorded up to the second actin meridional reflection, while the green arrow marks the region where the overall profile shapes remain consistent across models. Equatorial profiles are shown from relaxed and contracted actin filaments in panels (**A**) and (**B**), respectively. Profiles along the 6th layer line (59 Å) in the radial direction from relaxed and deformed actin filaments are shown in panels (**C**) and (**D**), respectively.

In contrast, the radial intensity profiles along the layer lines, even after normalization of grain sizes to atomic scale, differ between different coarse-grained models. Radial profiles along the equator of the patterns and at the 6th actin layer line at 59 Å are shown in Figure 11. On the equatorial profiles (Figure 11A,B), the 47-sphere model agrees with the all-atom case slightly above the 1st meridional reflection (~0.055 Å^−1^), while the 260-sphere model follows the all-atom profile slightly above the 2nd reflection (~0.08 Å^−1^). At the 6th layer line (Figure 11C,D), the 47-sphere model reproduces the profile up to the 1st maximum, whereas the 260-sphere model matches the all-atom model between the 1st and 2nd maxima (~0.06 Å^−1^).

**Figure 12:**
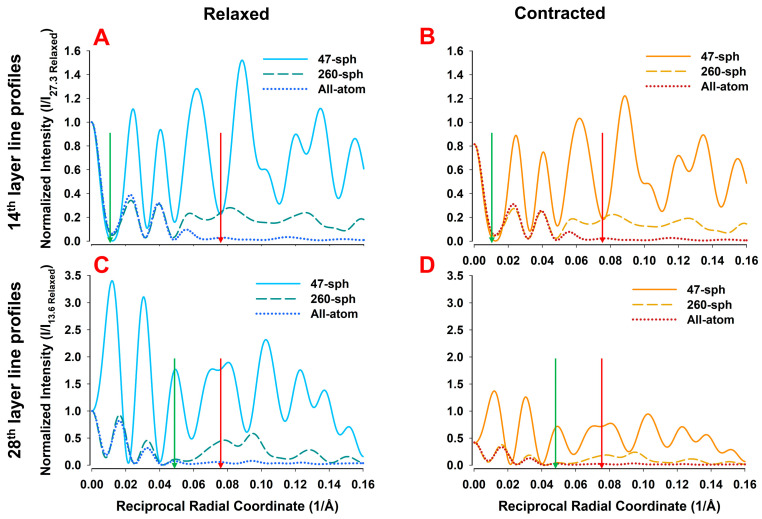
Comparison of diffraction profiles in the radial direction along the layer line of the 1st and 2nd actin meridional reflection for actin monomers represented by coarse-grained models with 47 spheres (solid lines), 260 spheres (dashed lines), and the all-atom model (dotted lines) in relaxed (**left**) and contracting (**right**) filaments. The intensities in the profiles are normalized to that of the first actin meridional reflection. The red arrows indicate the extent of observable reflections if experimental data are recorded up to the second actin meridional reflection, while the green arrows mark the region where the profiles remain consistent across different models. The radial profiles of the layer line corresponding to the 1st actin meridional reflection line in relaxed and contracting actin filaments are shown in panels (**A**) and (**B**), respectively. The radial profiles of the layer line corresponding to the 2nd actin meridional reflection from relaxed and contracted actin filaments are shown in panels (**C**) and (**D**), respectively.

Profiles along higher-order layer lines are presented in Figure 12. At the 14th layer line (Figure 12A,B), the 47-sphere model agrees with the all-atom system only up to ~0.01 Å^−1^, while the 260-sphere model maintains agreement up to ~0.045 Å^−1^. At the 28th layer line (Figure 12C,D), the 47-sphere model diverges extensively, whereas the 260-sphere model remains consistent with the all-atom results up to ~0.045 Å^−1^.

## 4. Discussion

### 4.1. Significance

The primary objective of this work was to provide a rigorous formulism for predicting X-ray fiber diffraction patterns from all-atom structural models of actin filaments, both at rest and under the nonuniform strain experienced during contraction. These predictions comprise detailed full 2D patterns that can serve as benchmarks for the types of structural information potentially obtainable from X-ray fiber diffraction experiments. In our previous studies [15,16], we showed that the meridional diffraction features from actin filaments contain valuable information regarding the local forces acting on myofilaments [15,16]. This information is required for detailed simulations of muscle using multiscale modeling aimed at translating local changes in molecular interactions, which will depend on local forces experienced by the molecules, whether caused by protein mutations or the effects of therapeutics, into functional changes at the organ level. In muscular organs, these changes manifest as changes to the actomyosin cycle, regulatory proteins, and auxiliary proteins such as titin and nebulin, all of which contribute to modulation of physiological muscle function. With detailed estimations of local strain at the myofilament level, these interactions can be simulated with much more confidence than with macroscopic measurements of tension on whole muscle fibers with their associated uncertainties. It should also be emphasized that this information would be hard to obtain any other way. Molecular dynamics (MD) has become a popular tool for studying the properties of actin in cells, e.g., [38]. While one can obtain global dynamic properties, e.g., [39], they do not address situations like this one where there are gradients of forces that accumulate along ~1 µm long thin filaments in the constrained geometry of the sarcomere. Similarly, MD has been used to study actin/myosin interactions [40] but, while they are likely to be useful when we know the strain distributions along the filament, they are unlikely to be helpful in studying these distributions on their own.

In this study, we extend our previous work by incorporating nonuniform strain into predictions of fiber diffraction patterns based on atomic-scale models of quasi-helical filamentous structures in thin filaments of striated muscle. While the focus of the work presented here was on actin filaments, the formulism developed here will be applicable to the myosin containing thick filaments as well in future planned work towards a general tool for predicting the diffraction patterns from striated muscle combining multiscale simulations and structural analysis.

In corroboration of our previous studies, predicted all-atom meridional profiles from actin showed very little effects of nonuniform strain on the first actin meridional reflection but with significant changes in peak shapes in the second-, third-, and higher-order meridional reflections (Figure 6), showing the potential value of higher-order reflections in assessing force heterogeneity in contracting muscle. With our new ability to calculate simulated fiber diffraction patterns considering all-atom filament structures for actin, we can now critically evaluate the adequacy of various coarse-graining approaches that have been used in the past for calculating diffraction features from actin, in particular the meridional reflections.

Our X-ray diffraction simulations indicate that the size of the coarse-grains will affect both the accuracy of intensities and the axial extent of observable reflections due to the grain size, which affects the form factor and associated spatial distribution of radii of the helical paths joining the grain centers. These combined effects can be seen in comparison with all-atom predictions with coarse-grained models with 47 and 260 spheres per actin monomer (Figure S7). The 47-sphere model was able to preserve the general profile of the first-order meridional reflection but failed to reproduce the intensities and peak shape profiles for the second- and higher-order reflections with sufficient accuracy when compared to the all-atom model, indicating that its effective reciprocal space resolution is limited to low spatial frequencies. In contrast, the 260-sphere model captures the first three meridional reflections with reasonable accuracy, although the fourth- and higher-order reflections begin to diverge from the all-atom predictions.

Despite the differences in the predicted intensities in the meridional reflections with varying degrees of coarse-graining (Figure 10), the profile widths due to nonuniform strain in the filaments were very similar, relatively insensitive to the degree of coarse-graining (Figure S7E,F). Thus, after normalization, the intensities of the outer meridional reflections to that of the first actin meridional reflection under resting conditions (Figure 10), the changes in the reflection profiles positions and shapes become comparable across coarse-grained models and to the all-atom model. These observations indicate that low-resolution coarse-grained models are adequate to estimate force distributions in actin filaments from the intensity distributions of the actin meridional reflections. Even a single sphere per monomer is sufficient in validation of the approach taken in previous studies [16]. Since these low-resolution predicted patterns can be calculated rapidly, they could, with further development, be used in an iterative process to fit observed experimental meridional patterns and extract the local force distributions as a routine procedure.

We also show here that the regions of reasonable agreement between the predictions of 2D X-ray patterns from the 47- and 260-sphere coarse-grained and all-atom models are understandable in terms of the resolution imposed by the size of spheres used in the coarse-grained models. These limitations arise from two main effects, namely, (i) the intensity transform of the coarse-grains attenuated by the form factor and (ii) the reduced number and scattered distribution of the radii of helical paths through the coarse-grains. While it might appear that the limiting resolution might be simply the average equivalent sphere radius, $r_{s}$, it is lower than that due to the shape of the Fourier transforms of the spheres where the first zero in the transform is at substantially lower resolution (Figure 8, Table 1) than $r_{s}$. Due to the strong curvature of the transform approaching the zeros in the transforms, predicted intensities at reciprocal radii approaching these zeros will be substantially less than with the all-atom predictions leading to the observed discrepancies between coarse-grained and all-atom model predictions (Figure 10, Figure 11 and Figure 12). The minimum sphere radius imposed by the grain size also has strong effects on the radial and equatorial profiles (Figure 10, Figure 11 and Figure 12). While the 47-sphere model deviates significantly beyond the first intensity maximum, the 260-sphere model maintains good agreement with the all-atom case up to higher reciprocal radial coordinates.

There have been few attempts to interpret changes in the higher-order actin layer lines (past the strong 6th and 7th actin layer lines), since, historically, they have been difficult to record, but this could change in the near future with the availability of higher resolution and better resolved X-ray fiber patterns using 4th generation synchrotron sources. All-atom model predictions yield very rich diffraction patterns (Figure 5) with relatively strong diffraction features near the actin meridional reflections. Here, we show that the 260-sphere model can match the predictions of the all-atom models up to a reciprocal radial coordinate of ~0.05 A^−1^, which includes the first two intensity maxima on layer lines close to the first and second actin meridional reflections which have been observed experimentally (e.g., Figure 4, a key quantitative finding of this study).

### 4.2. Computational Considerations

Given the limitations of coarse-grained models, why not always carry out all-atom simulations? Our long-term goal is to be able to efficiently simulate the structural changes in series of 100–1000 images from time-resolved X-ray diffraction experiments. This would allow direct comparison with MUSICO predictions of the changing sarcomere protein configurations as a function of the force and length variations during an experiment. The MUSICO simulations and the predicted diffraction patterns would be refined against observed diffraction patterns to produce a sequence of structural models of sarcomere protein configurations consistent with the force and length changes during a time-resolved experiment. In such a process, the time to calculate each predicted pattern is a significant limiting factor.

Using a Microsoft Windows desktop system using a single core (Intel Core i9 with 32 GB), simulating a single azimuthal angle for an actin filament containing ~2991 atoms takes 9 h with 18 azimuthal angles, 7 days. On a dedicated high memory node in our high-performance computing cluster (HPC) (comprising 4 Intel Xeon Gold 6338 N 2.2 GHz thirty-two-core processors and 2048 GB RAM) with 128 processor cores, recent optimizations reduce per-atom execution time to ~20 s, enabling one azimuthal angle to be computed in ~8 min, and 18 angle approximations to a full 3D cylindrical transform in ~2.5 h, more than 60× faster than on a single-core machine.

Significant reductions in computing time can be realized with even modest degrees of coarse-graining. At one extreme, if only the meridional axial intensity profiles are of interest, we have shown that we can approximate each actin monomer as a single sphere and one azimuthal angle (all that is needed) can be calculated in 20 s. Calculations of the 2D pattern for the 260-sphere model, however, at one azimuthal angle on a single-core machine, takes ~45 min (~14 h total for 18 angles). In contrast, on our 128-core HPC node, the entire 18-angle set can be completed in ~13 min. This degree of throughput would enable, for example, generating patterns for 100 simulation frames in ~21 h on the HPC system, comparable to the length of time required for MUSICO mechanical Monte Carlo simulations of the molecular configurations of 500 myosin filaments and 1000 actin filaments during a contraction cycle required to match the experimental force and length changes. While additional improvements can be expected with GPU implementations, the time required for computing the 260-sphere coarse-grained model with existing CPU-based clusters will not be prohibitive for satisfactory simulation of the actin patterns found in typical fiber diffraction patterns from muscle.

## 5. Materials and Methods

### 5.1. Sarcomere Lattice Geometry and Actin Filament Configuration Under Relaxed and Contracting Conditions

Myosin and actin filaments are arranged in a hexagonal lattice (Figure 2A) within the sarcomere, the fundamental contractile unit of vertebrate striated muscle [35]. Each myosin filament is decorated with crowns of myosin dimers, spaced by ~14.3 nm apart along the filament, and each crown consists of three myosin dimers arranged with transverse orientations spaced by 120°, with successive crowns rotated by +40° when viewed towards the Z-line [27]. The actin monomers in each thin filament form a double-helix complexed with the regulatory proteins tropomyosin and troponin, with binding sites spaced ~5.5 nm apart on each strand, with helical a half-period of ~35.8 nm [41]. In relaxed muscle, actin filaments exhibit a monomer spacing of ~2.74 nm, which corresponds to ~5.47 nm on each strand and with a half period of ~35.57 nm [15,16,28,29]. The 3D sarcomere geometry with extensible filaments, myosin head binding domains, and actin binding sites, requires alignment in both the longitudinal positions along the filament and the angular positions in the azimuthal plane [27]. A myosin head and the closest actin site form the most probable pair that can create a crossbridge, interconnecting the actin and myosin filaments. During contraction, bound myosins and other sarcomere structures such as titin and myosin binding protein C (MyBP-C) deform both myosin and actin filaments. This deformation is nonuniform along the filaments and it is reflected in nonuniformly deformed helices.

**Figure 13:**
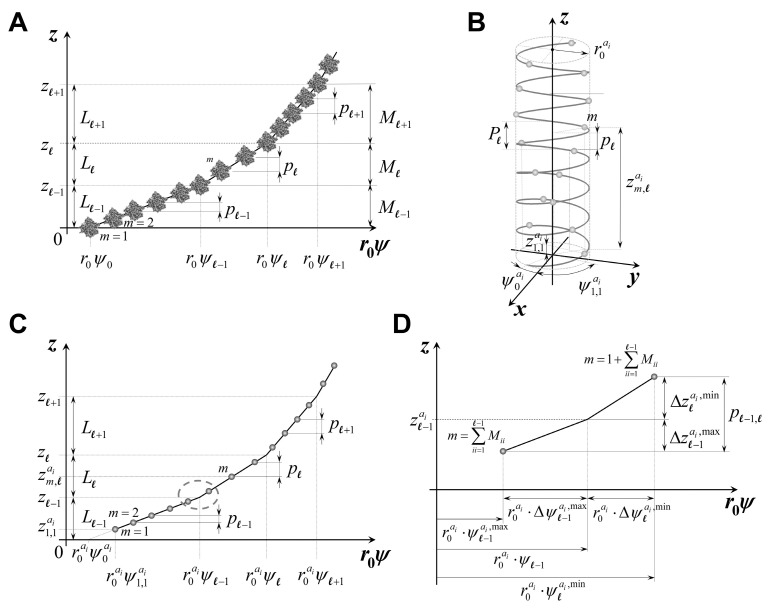
The geometry of a nonuniformly deformed discontinuous helix. (**A**) Three consecutive segments of a nonuniformly deformed discontinuous helix, represented by a set of helically arranged subunits, are displayed in the radial projection as the miniature dark gray monomers connected with straight lines. Changes in the axial monomer spacings are defined at positions, $z_{l}$, of the planes where crossbridges are bound to actin filament. The axial positions of sequential planes define the segments with the constant pitch, $P_{l}$, over the segment length, $L_{l}=z_{l}-z_{l-1}$, where index, l, denotes the current number of the segment. The index of the coordinate of each segment end point, i.e., the coordinate where the pitch, i.e., slope, changes is set to coincide with the current segment number, l. The changes in actin subunit spacings, $p_{l}$, follow the segment slopes, and the number of subunit spacings, within a segment, $L_{l}$, is denoted as $M_{l}$, thus $L_{l}=M_{l}\cdot p_{l}$. The number of these constant pitch segments is $l_{\max}$. Therefore, for each actin filament, there will be $l_{\max}+1$ coordinates $z_{l}$, where $z_{0}$ is the coordinate of the tip of each actin filament, denoted to be equal to 0, and $l_{\max}$ coordinates $z_{l}$ measured from the tip of the actin filament to the position of the planes where crossbridges are bound to the actin filament, except $z_{l_{\max}}$, which is the coordinate of the plane at the boundary with Z-disk. Note that the index of the plane number, l, coincides with the segment length and pitch indices because the position of the first plane is calculated from the tip of actin filament, i.e., from coordinate $z_{0}$. The discontinuous helix is defined by the radius of the helix cylinder, $r_{o}$, and angular position of the helix passing through the load plane, $\psi_{l}$. (**B**) Each atom, $a_{i}$, in a subunit forms its own discontinuous helix where the atom’s positions arranged along the helix are shown as gray spheres. The position of the atom, $a_{i}$, in the subunit is defined by radial position $r_{0}^{a_{i}}$, assumed to be the same in all subunits. The axial coordinate of the atom, $a_{i}$, in all subunits, $z_{m,l}^{a_{i}}$, measured from the zeroth plane (l=0) and the azimuthal angle $\psi_{m,l}^{a_{i}}$, where index m is the current number of a subunit and l is the segment number. (**C**) Radial projection of a nonuniformly deformed discontinuous helix passing through an array of positions of the atom, $a_{i}$, shown as gray spheres. The change in spacing, $p_{l}$, for atom, $a_{i}$, is shown through radial projection coordinates $z_{m,l}^{a_{i}}$ and $r_{o}^{a_{i}}\psi_{m,l}^{a_{i}}$. Depending on a position of the atoms within the first monomer ($z_{1,1}^{a_{i}}$ and $\psi_{1,1}^{a_{i}}$) and following the assumption that crossbridge force is transferred to the actin in a single plane perpendicular to the helix axis at $z_{l}$, the positions of atoms rarely coincide with the positions of the planes, $z_{l}$. Thus, for each atom, $a_{i}$, the spacing, $p_{l}$, is constant for the subunits within the segment, l, except where the spacing shares attributes of two segments, e.g., of l−1 and l as shown in the gray dotted circle. (**D**) The zoomed in detail in the gray dotted circle (from (**C**)). The spacing between the two positions of the atom, $a_{i}$, spanning two segments is defined by the slopes in segments l−1 and l. The contribution fractions of l−1 and l segment slopes for the intersegment atom, $a_{i}$, spacing, $p_{l-1,l}^{a_{i}}$, are defined by $\Delta z_{l-1}^{a_{i},\max}$ and $\Delta z_{l}^{a_{i},\min}$, and pitches $P_{l-1}$ and above the load plane, $z_{l}^{a_{i},\min}$, in the same region but in the following monomer $P_{l}$.

A nonuniformly deformed discontinuous helix can be viewed as a sequence of axial segments, each with constant monomer spacing and a corresponding local pitch. These pieces are joined together so that the filament can accommodate changes in monomer spacing introduced by cross-bridge forces or other perturbations. For each segment, the helical path is defined by the helix radius, the azimuthal position of the atom (or grain) in the first monomer, and the local axial separation between consecutive sampling planes. Connecting these segments generates a complete discontinuous helix that follows the deformation of the filament (Figure 13). The same construction applies to all atoms or coarse-grained spheres, producing a family of discontinuous helices that collectively describe the deformed filament geometry. Additional geometric details are provided in Appendix A.

### 5.2. Discontinuous Helices Derived from All-Atom Actin Filament Structure Under Relaxed and Contracted Conditions

In the relaxed filament, the axial spacing between monomers is virtually uniform and any minor nonuniformity caused by thermal fluctuations can be neglected. In the all-atom formulation, each atom therefore traces a discontinuous helix characterized by a fixed radius and constant axial step. The helical path is fully determined by the atom’s position in the first monomer, from which identical axial and angular increments are applied throughout the filament (Figure 13B,C). This produces a family of regularly spaced discontinuous helices that collectively reproduce the periodic atomic structure of the relaxed actin filament.

Under contracting conditions, the filament experiences nonuniform axial deformation, and the spacing between monomers varies along its length. To represent this configuration, the filament is divided into short axial segments, each with its own characteristic monomer spacing and corresponding helical pitch. Within each segment, atoms follow discontinuous helices with constant local spacing, while transitions between adjacent segments introduce small discontinuities in axial position. The angular progression of each atom is assumed to remain unchanged relative to the relaxed configuration, allowing deformation to be captured entirely through changes in local pitch and intersegment spacing. This piecewise construction generates a set of discontinuous helices that reflect the nonuniform strain distribution in the contracted filament. A detailed mathematical description of the coordinate definitions, segment indexing, axial and angular increments, and expressions for fragment contributions under deformation are provided in Appendix B and Appendix C.

In the segmented model described above, each axial segment has clearly defined boundaries that mark the first and last monomers belonging to that segment. These boundaries determine how the filament deformation is distributed along the length of the helix and how atoms transition from one segment to the next, especially near the loading planes. Because discontinuous helices are sampled at discrete monomer positions, it is useful to refer to these boundary locations when describing atom positions in segments adjacent to a loading plane. We therefore introduce upper and lower axial boundary coordinates for each segment, which allow us to describe how atoms are positioned relative to the segment boundaries under deformation.

**Figure 14:**
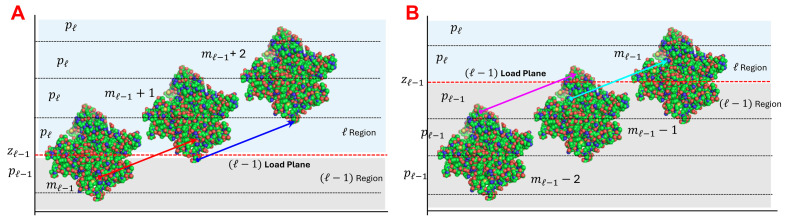
The position of the atoms $a_{i}$ in actin monomers below and above the load plane. There are five distinct regions within the monomer where atom $a_{i}$ can be located. Depending on which region the atom resides in, different monomers can be associated with the calculation of the intersegment spacing $p_{l-1,l}^{a_{i}}$, except when the atom $a_{i}$ is in the proximity (±ε) of the load plane, i.e., region (i). (**A**) When the coordinate $z_{l-1}^{a_{i},\max}$ atom $a_{i}$ is located below the load plane and threshold region (i) but not lower than $-p_{l}^{a_{i}}$, it is assigned to region (ii) and if $z_{l-1}^{a_{i},\max}$ is further below the load plane, i.e., more than $-p_{l}^{a_{i}}$, it belongs to region (iii). Atoms $a_{i}$ with $z_{l-1}^{a_{i},\max}$ in the region (ii) are associated with monomer $m_{l-1}$, while those in region (iii) are assigned to monomer $m_{l-1}+1$. (**B**) When the atom $a_{i}$ with coordinate $z_{l-1}^{a_{i},\max}$ is the above load plane but not exceeding $p_{l}^{a_{i}}$, it belongs to region (iv), and if it lies beyond $p_{l}^{a_{i}}$, it is in region (v). Atoms $a_{i}$ with $z_{l-1}^{a_{i},\max}$ in the region (iv) are associated with monomer $m_{l-1}-1$, whereas those in region (v) are assigned to monomer $m_{l-1}-2$. The arrows represent the vector between the atom $a_{i}$ coordinate below the load plane, $z_{l-1}^{a_{i},\max}$, and above the load plane, $z_{l}^{a_{i},\min}$, in the same region but in the following monomer. Red dashed lines denote the load plane l−1. Gray background represents l−1 region, while blue background represents region l above the load plane l−1.

Atoms located near a loading plane may lie in different monomers depending on their precise axial and angular positions. To account for this, we distinguish five positional regions relative to each segment boundary, as illustrated in Figure 14. These regions determine whether an atom is assigned to the monomer just below the loading plane, the monomer just above it, or another monomer whose axial coordinate falls within the spacing interval surrounding the boundary. Correctly identifying these five regions is essential for computing the Fourier transform of nonuniform discontinuous helices—whether represented at full atomic resolution or through coarse-grained spheres—because the scattering contribution of each atom or grain depends on assigning it to the correct monomer index in the deformed configuration.

Formal definitions of these five positional regions, together with the corresponding rules for monomer assignment and expressions for the upper and lower segment boundary coordinates, are provided in Appendix C. These definitions also form the basis for calculating the intersegment spacing $p_{l-1,l}^{a_{i}}$ and the monomer index transitions used in the Fourier analysis. The computation of the coordinate $z_{m,l}^{a_{i}}$ of atom $a_{i}$ in monomer m within segment l is given in Appendix D and Appendix E.

### 5.3. Fourier Transform of a Nonuniformly Deformed Discontinuous Helix

The Fourier transform of a discontinuous helix of an atom $a_{i}$ that is nonuniformly deformed has nonzero values at $z^{a_{i}}\left(m,p_{l},p_{l-1,l}^{a_{i}}\right)$, i.e., at the spatial positions of the atom $a_{i}$ in monomers along actin in the deformed configuration. Note that in deformed configuration, there are two types of intermonomer spacings: within the segments, $p_{l}$, which are the same for all discrete helices, and intersegment monomer spacings, $p_{l-1,l}^{a_{i}}$, which are specific for each discrete helix, depending of the position of atom $a_{i}$ within the monomer, and size of spacing fragments and associated strains on each side of the load plane, l−1. Detail derivation of $z^{a_{i}}\left(m,p_{l},p_{l-1,l}^{a_{i}}\right)$ is shown in Appendix D.

Given these definitions, the Fourier transform, ${\hat{f}}^{a_{i}}\left(R,\Psi ,Z\right)$, may be defined for a discontinuous helix of infinitesimal small units passing through atom $a_{i}$ a set of planes perpendicular to z axis $\rho_{k}$ at the positions of atoms $a_{i}$. For the actin filament of finite length containing $m_{\max}$ monomers, the planes are defined by $\rho_{k}^{a_{i}}=\sum_{m=1}^{m_{\max}}\delta \left[z-z^{a_{i}}\left(m,p_{l},p_{l-1,l}^{a_{i}}\right)\right]$. The full expression of the Fourier transform of a discontinuous helix of atom $a_{i}$ that is nonuniformly deformed, in terms of atom coordinates $r_{0}^{a_{i}}$, $\psi_{m,l}^{a_{i}}$ and $z_{m,l}^{a_{i}}$, for nonzero values at $z^{a_{i}}\left(m,p_{l},p_{l-1,l}^{a_{i}}\right)$ is then defined as

(1) $$\begin{array}{ll} {\hat{f}}^{a_{i}}\left(R,\Psi ,Z\right) & =\frac{r_{0}^{a_{i}}}{{\left(2\pi \right)}^{1/2}}\sum_{n=-\infty }^{+\infty }e^{in\cdot \left(\Psi +\frac{\pi }{2}-\psi_{1,1}^{a_{i}}\right)}\cdot J_{n}\left(2\pi r_{0}^{a_{i}}R\right)\\ & \cdot \left\{p_{1}\cdot \sum_{m=1}^{M_{1}}e^{-in\cdot \left(m-1\right)\Delta \psi_{o}^{a_{i}}}\;e^{i2\pi \cdot Z\cdot z_{m,1}^{a_{i}}}\right.\\ & +\sum_{l=2}^{l_{\max}}\left[p_{l}\sum_{m=2+\sum_{l=1}^{l-1}M_{l}}^{\sum_{l=1}^{l}M_{l}}e^{-in\cdot \left(m-1\right)\Delta \psi_{o}^{a_{i}}}e^{i2\pi \cdot Zz_{m,l}^{a_{i}}}\right]\\ & +\sum_{l=1}^{l_{\max}-1}\left[p_{l,l+1}^{a_{i}}\cdot e^{-in\cdot \left(m-1\right)\Delta \psi_{o}^{a_{i}}}\cdot e^{i2\pi \cdot \cdot Zz_{1+\sum_{ii=1}^{l}M_{ii},l}^{a_{i}}}\right]\\ & \left.+\left[p_{l_{\max}}\cdot e^{-in\cdot \left(m-1\right)\Delta \psi_{o}^{a_{i}}}\cdot e^{i2\pi \cdot Z\cdot z_{1+\sum_{l=1}^{l_{\max}}M_{l},l_{\max}}^{a_{i}}}\right]\right\} \end{array}$$

The Fourier transform of the discontinuous helix of finite size atoms, $a_{i}$, is represented as the product of the transform of the discontinuous helix of finite length and the transform of the finite atom size object, $\rho_{s}^{a_{i}}$. The atom size and electron density are obtained for each atom as its Van der Waals radius, $r_{vdw}^{a_{i}}$, from the chosen actin atomic structure (dom4b.pdb). From $r_{vdw}^{a_{i}}$, the effective atomic volume $V_{eff}^{a_{i}}=0.34\cdot \left(4/3\right)\pi \cdot {\left(r_{vdw}^{a_{i}}\right)}^{3}$ is calculated. For coarse-grained structures, the equivalent sphere radius is calculated from the volume of all atoms in the coarse-grained structure (Figure S1). Finally, the Fourier transform of actin filament is represented by a sum of the transforms over all discontinuous helices of a finite length associated with atoms $a_{i}$ and the transforms of their finite (atom) sizes (Equation (1)):

(2) $$\hat{f}\left(R,\Psi ,Z\right)=\sum_{i=1}^{N_{a_{i}}}{\hat{f}}^{a_{i}}\left(R,\Psi ,Z\right)\cdot G_{s}^{a_{i}}$$

where $N_{a_{i}}$ is number of atoms in the actin monomer, containing about 2991 atoms. For the coarse-grained structures, we used the same methodology but with spheres of equivalent radii and equivalent electron densities (mass).

The 3D Fourier transform (Equation (2)) is cylindrically symmetric about the meridian, but the phase oscillates azimuthally, depending on the Bessel order. Since the observed X-ray diffraction patterns are recorded as the cylindrically averaged diffracted intensity, it is necessary to calculate average intensity from integral of local intensities over azimuthal angles, Ψ, from 0 to 2π. This procedure is described in Appendix E.

### 5.4. Actin Monomer Atomic Structure

Helical polymers, such as actin filaments, typically consist of subunits arranged in a helical pattern. In the case of actin filaments, the subunits are the G-actin monomers organized into a helical F-actin configuration. The atomic coordinates for actin monomer structures are available from several Protein Data Bank (pdb) entries, including 1ATN.pdb [17,42], 3MFP.pdb ([43]; containing five monomers), and 2ZWH.pdb [44]. The general formulation of all-atom models for simulating X-ray diffraction patterns permits use of any of the above or other available actin monomer structures. In the present study, we have used the structure contained in dom4b.pdb (see Supplementary Materials), which was provided through personal communication with K. Holmes. This particular structure is a modified version of the 1J6Z.pdb structure from [45] and has been chosen because it originates from a three-monomer F-actin assembly (3actin.pdb) and was also used in a five-monomer model developed by Holmes’s group [36]. Unlike coordinate sets derived from isolated G-actin (e.g., 1ATN.pdb) or from later cryo-EM-based F-actin reconstructions (e.g., 3MFP.pdb from Fujii et al. [43]), the dom4b.pdb structure was refined within filament-level assemblies and optimized to match fiber X-ray diffraction data. A closely related monomer was already employed by Holmes et al. in their early all-atom simulations of F-actin fiber diffraction patterns using their “Fiber” program [17], in which atomic models were iteratively refined to reproduce experimental relaxed layer-line intensities using the helical selection rule and corrections for orientational disorder.

Compared to cryo-EM models such as 3MFP.pdb, which benefit from direct visualization of secondary structures at 6.6 Å resolution, fiber diffraction-based models (such as those of Holmes [36] or Oda [44]) are inherently limited by cylindrical averaging and model-dependent fitting. Fujii et al. [43] also highlighted local conformational differences between their model and Holmes’s, especially in flexible regions such as loops and in the relative orientations of actin domains. As they noted, diffraction-based methods constrain atomic refinements within narrow bounds and may produce unnaturally tight packing when simulated in vacuum. While we acknowledge that newer cryo-EM-derived structures offer greater local accuracy, the dom4b.pdb model remains appropriate for our current purposes to demonstrate a general all-atom simulation framework, which can readily incorporate updated actin monomer structures in future applications.

### 5.5. Coarse-Grained Actin Monomer Structure

All-atom simulations demand significant computational resources and extended execution times. Moreover, these high-resolution images often surpass the resolution of experimentally observed patterns. A more practical approach involves using a coarse-grained representation of the atomic-level molecular structure [31], which may suffice to allow interpretation of the majority of experimental X-ray diffraction data, providing that the coarse-graining does not result in excessive loss of information. While those models captured low-order reflections effectively, the use of uniform-radius spheres introduced an additional modulation of intensities due to the sphere form factor.

To avoid these artifacts and more accurately evaluate the effects of coarse-graining, we developed coarse-grained models in which the actin monomer is represented by a collection of grains, represented as equivalent spheres whose volumes approximate the local electron density distribution of the about equal fragmented actin monomer parts at varying resolutions (Figure S1), while preserving the total atomic mass. The sizes of these spheres are determined by a clustering procedure applied to the atomic coordinates of the monomer producing spheres of varying effective volumes. This introduces variable grain sizes with differences in radius of no more than 10% (Figure S3), which exert only minor effects on the simulated X-ray diffraction patterns.

### 5.6. Clustering Procedure

We begin by partitioning the atomic coordinates (in Cartesian space) of the actin monomer into a predetermined number of clusters (e.g., 1, 9, 47, 260, and 1016 clusters) using custom MATLAB code (MATLAB R2017b). This code implements the k-means clustering algorithm, enhanced by the k-means++ heuristic to determine initial centroid positions, which improves both the convergence speed and the quality of the final clustering solution [46,47]. For each specified cluster count, the clustering is repeated 100 times. In each iteration, atoms are assigned to clusters based on their proximity to cluster centroids, and the centroid of each cluster is recalculated iteratively. The cluster volume is then computed as the cumulative volume of the atoms contained within each cluster. We calculate the standard deviation (or standard error, depending on the number of clusters) of these volumes across the clusters for each iteration. The iteration yielding the minimum variability is selected as the optimal configuration, and its corresponding cluster centers are used in the coarse-grained model.

### 5.7. Transformation and Grain Size (Equivalent Sphere) Parameters

Following the clustering, the Cartesian coordinates (*X*, *Y*, *Z*) of the optimal cluster centers are transformed into cylindrical coordinates to obtain the radial distance, $r_{o}^{cc_{i}}$, from the actin filament’s main axis, the angular position, $\psi_{o}^{cc_{i}}$, and axial position, $z_{o}^{cc_{i}}$. These cylindrical coordinates are used to position the corresponding grains in the model. The grain sphere radius for each cluster is then determined based on its calculated volume, reflecting the local electron density distribution. The Mean grain sphere radii obtained from our procedure for various models are as follows: (a) 1-sphere model: Mean radius of 22.39 Å; (b) 9-sphere model: Mean radius of 10.75 ± 0.44 Å (compared to a previously reported value of 10 Å [31]); 47-sphere model: Mean radius of 6.2 ± 0.27 Å (previously reported as ~6 Å [31]); 260-sphere model: Mean radius of 3.48 ± 0.33 Å, yielding a resolution sufficient to capture reflections beyond the fourth actin meridional reflection (6.8 Å); and 1016-sphere model: Mean radius of 2.18 ± 0.32 Å, which is close to the Mean atomic radius in the actin monomer (1.55 ± 0.15 Å). This level of granularity, representing roughly one-third of the total atoms, permits the detection of high-resolution features beyond those observed in previous studies (e.g., the 3.3 Å resolution reported by [44]).

### 5.8. Model Parameters for MUSICO Simulations

#### 5.8.1. Sarcomere Geometry and Myofilament Elasticity

In the bullfrog sartorius muscle 3D sarcomere lattice, actin filaments are ~1 µm long, with 364 monomers in total [48]. The actin monomer spacing is 2.736 nm and a half period of one strand is 35.57 nm [15,28,29]. The length of a myosin filament is ~1.58 µm, with 50 crowns, i.e., 150 myosin molecules per half-thick filament, with crown spacing of 14.3 nm [49,50]. The sarcomere length is set at 2.17 µm to be in a full overlap and the lattice inter-filament spacing of frog sartorius muscle is $d_{10}=36.5$ nm [51,52]. For the calculation of azimuthal angles, which define relative position of myosin dimer to the correct orientation of a binding site on actin filament, we used an actin filament radius $r_{a}=3.5$ nm and thick filament backbone radius $r_{m}=7.9$ nm [27]. For simplicity, we limited all stochastic simulations to a half sarcomere with 500 myosin and 1000 actin filaments. This number of filaments is comparable to the number of filaments in a cross section of a typical myofibril and provides sufficient statistical averaging without running the simulation multiple times.

Actin and myosin filaments are extensible with filament moduli (elastic modulus times cross section area) derived from X-ray diffraction or direct measurement: for actin, $K_{a}=0.65\times 10^{5}$ pN [53], and for myosin $K_{m}=1.32\times 10^{5}$ pN [28].

#### 5.8.2. Nine-State Crossbridge Cycle Model Parameters

Following the comprehensive approach of Smith et al. [32,33], the state transition rate constants are, for binding, $\Delta G_{bind}=-4.8\;k_{B}T$ that corresponds to the equilibrium constant is $K_{bind}=k_{bind}/k_{unbind}=e^{-\Delta G_{bind}/k_{B}T}≅125$ and forward rate constant at zero crossbridge strain is $k_{bind}=200$ s^−1^; for the first power stroke, equilibrium constant is defined by $\Delta G_{stroke}=-3.9\;k_{B}T$, power stroke $d_{1}=5.5$ nm and forward rate constant at zero crossbridge strain is 7500 s^−1^; for the second power stroke, equilibrium constant is defined by $\Delta G_{stroke}=-6.2\;k_{B}T$, power stroke $d_{2}=3.5$ nm and forward rate constant 1.5 × 10^5^ s^−1^; equilibrium Pi release constant $K_{Pi}=k_{Pi+}/k_{Pi-}=10$, where $k_{Pi+}$ is after the first power stroke equal to 80 s^−1^ and after the second 400 s^−1^; and for ADP release/detachment, $k_{ADP}^{0}=100$ s^−1^ and second power stroke δ=1 nm. In all simulations, crossbridge stiffness is taken to be κ=1.3 pN/nm (as used by Mijailovich et al. [16]) and the value for $k_{B}T=3.91$ pN·nm at 10 °C or 283.15 °K [29].

### 5.9. Normalization of Isometric Tension Force per Thin Filament

The spatially explicit model in MUSICO calculates the force in each actin and myosin filament, and the force is directly related to the stochastic kinetics of the actomyosin cycle and the elasticity of crossbridges and myofilaments. The simulations are performed by setting up the initial conditions and letting the system evolve over time (for example, 0.5 s with 10 µs steps). Due to the stochastic process of myosin interactions with actin, the forces in the myofilaments fluctuate in time and each filament experiences somewhat different force [16]. Overall, each simulation with 500 myosin filaments per half sarcomere provides not only sufficient averaging of the muscle fiber tension but also the fluctuations in force in each myosin and actin filament. The scales between the filament force and muscle tension are related by a factor that takes into account how many myosin filaments there are per unit of the fiber cross sectional area. In intact fibers from frog skeletal muscle, the fraction of cross section occupied by myofibrils, for a lattice spacing of $d_{10}=36.5$ nm at a sarcomere length of 2.17 µm, is 0.83 [54]. Here, the calculated average force per thin filament, ${\bar{F}}_{a}$, is 315 pN, which corresponds to a muscle isometric tension, $T_{0}$, of 340 kPa.

## 6. Conclusions

### 6.1. Summary

In summary, we now have a rigorous formulation for the calculation of 2D diffraction patterns from helical structures under nonuniform strain, with specific application to the actin-containing thin filaments in striated muscle, which could be applicable to any fibrous protein system under nonuniform strain. We also have shown how much information is being lost with various degrees of coarse-graining and discuss this in the context of the kinds of diffraction patterns currently obtainable experimentally. We show that simple coarse-grained models that can be calculated quickly are adequate for estimating force distributions in actin filaments from the peak positions and peak widths of meridional reflections. This will be particularly important for time-resolved studies. In contrast, higher-resolution coarse grained models (260 spheres per actin monomer) are required to adequately predict layer-line intensity distributions for comparison with experimental diffraction patterns up to a resolution of ~0.05 A^−1^, a resolution greater than typically achieved with current muscle diffraction experiments. Higher resolution predictions may be obtained, when needed, using all atom calculations.

### 6.2. Limitations of the Current Studies

Our computational approach has a number of limitations. In the formulation presented here, the possibility of twisting of the filament involving unwinding of filament helices is not considered but this situation could be straightforwardly added to the analysis in the context of simulating experimental data that suggests such unwinding. Hydrogen atoms were not included in our calculations since they have little mass. Even in macromolecular crystallography, very high-resolution data or special refinement procedures are necessary to detect hydrogens and are unlikely to affect predictions at the lower resolutions typically encountered in fiber diffraction experiments. Cylindrical averaging is inherent in fiber diffraction studies, leading to loss of information. To calculate, this rigorously requires, in principle, integrating continuously throughout 360 degrees, which is computationally infeasible. However, comparison of predictions assuming different numbers of integration angles, as shown in Figures S8 and S9, demonstrates that, at the resolutions we are considering, even the 18 angles used here are more than enough to adequately sample the cylindrically averaged intensity transform. Additional factors need to be considered in the context of comparing predictions to specific experimental data sets. In addition to instrument-specific factors, these include considerations of disorder, both in the myofilament lattice and in the individual filaments, which will be the primary determinant of what reflections are observable experimentally. These effects will be addressed in future publications, currently in preparation, where predictions are compared to specific sets of experimental data.

### 6.3. Outlook

Next steps in this process will be the extension of these techniques to regulated thin filaments, containing troponin and tropomyosin in addition to actin. Work will then be extended to the thick filaments containing myosin and accessory proteins including titin and myosin binding protein C, as informed by ongoing cryo-electron microscopy studies. Successful implementation of these techniques will be foundational towards our long-term goal of predicting full 2D X-ray fiber diffraction patterns from striated muscle, in combination with spatially explicit MUSICO simulations of sarcomere structures during active contraction, as an interpretive tool for existing and future static and time-resolved X-ray diffraction experiments on muscle.

## Figures and Tables

**Table 1:** The effect of the coarse-grain size, defined by the radius of the equivalent sphere, on the radial position where the Fourier transform (FT) of actin filament diffraction patterns exhibits rapid intensity decay in proximity of its first zero value.

Coarse-Grained Model	Average Equivalent Sphere Radius (Å)	First Zero of the 3D FT of Sphere (Å)
Single Sphere	22.39	31.32
9 Spheres	10.75	15.03
47 Spheres	6.20	8.67
260 Spheres	3.48	4.87
1016 Spheres	2.18	3.05
All Atom	1.55	2.17

## Data Availability

The original contributions presented in this study are included in the article/Supplementary Material. Further inquiries can be directed to the corresponding authors. The MUSICO modeling computational platform is proprietary to FilamenTech Inc. Source code of programs to calculate the diffraction from discontinuous deformed helices can be provided upon request to the corresponding authors.

## Supplementary Materials

The following supporting information can be downloaded at: https://www.mdpi.com/article/10.3390/ijms27010280/s1. Actin monomer structure: dom4b.pdb, Supplementary material text with figures.

## Abbreviations

2-D	Two-Dimensional
3-D	Three-Dimensional
Cryo-EM	Cryo-Electron Microscopy
FT	Fourier Transform
GPU	Graphics Processing Unit
HPC	High-Performance Computing
MUSICO	Muscle Simulation Code
PDB	Protein Data Bank
sph	sphere

## Appendix A

### Appendix A.1. Basic Principles in Constructing Discontinuous Helices for Specified Atoms in a Helical Molecular Structure

The vertebrate sarcomere lattice of actin filaments contains 340–460 helically arranged actin subunits. The diffraction patterns of actin filament can be represented as the Fourier transform of multiple discontinuous helices, with one helix corresponding to each atom in the actin filament monomer assembly. The basic principles used to construct these structures are illustrated in Figure A1. In muscle cells, filaments are typically arranged as a helical array of multi-atom monomers where each atom, $a_{i}$, within the monomer has its own helical wire, $\rho_{h}^{a_{i}}$, passing through the same atom along all F-actin monomers. A cylindrical helix is a smooth 3D curved line such that any point on this helix is equidistant from a fixed line in space, called the helix axis, and its tangent makes a constant angle with the axis. Therefore, in cylindrical coordinates, the helix axial coordinate, z, is proportional to angle ψ, thus, for the axial increment of Δz equal to one pitch, P, the angle increment Δψ is equal to 2π. In a relaxed actin filament, the helix has a constant pitch, $P_{o}$. The equation of the helix passing through atom $a_{i}$ is defined as $r=r_{o}^{a_{i}}$, where $r_{0}^{a_{i}}$ is radius of the helix cylinder of and $\psi_{o}^{a_{i}}$ is the angle at which the helical wire intersects the plane perpendicular to the longitudinal axis at the tip of the actin filament, $z=z_{o}=0$ (Figure 3C).

The atoms $a_{i}$ on this helical wire are represented as a discontinuous helix, $\rho_{h,k}$, which can be constructed as the product of a continuous helical wire, $\rho_{h}$, with a pitch P, and a set of horizontal density planes, $\rho_{k}$. These planes pass through a selected atom $a_{i}$ over all subunits, the planes are axially separated from the next, in a sequence by a distance p [30]. In relaxed actin filaments, the axial separation between atoms also has a constant value, $p_{o}$. By repeating the process of forming discontinuous helices for all atoms in an actin monomer (Figure 3), one can generate a set of discontinuous helices that collectively represent the periodic structure of the actin filament (Figure 3C). Each discontinuous helix, $\rho_{h,k}^{a_{i}}$, for each atom, $a_{i}$, or $\rho_{h,k}^{cg_{i}}$, for each grain, $cg_{i}$, is defined by $r_{o}^{a_{i}\left(or\;cg_{i}\right)}$, angular coordinate $\psi_{o}^{a_{i}\left(or\;cg_{i}\right)}$ where the helix passes through the plane at axial coordinate $z_{o}^{a_{i}\left(or\;cg_{i}\right)}=0$, the atom $a_{i}$ axial coordinate in the first monomer, $z_{1,1}^{a_{i}\left(or\;cg_{i}\right)}$, the atom (or grain) separation, $p_{o}$, and the angular increment between atoms $a_{i}$ in sequential monomers, $\Delta \psi_{o}^{a_{i}\left(or\;cg_{i}\right)}=\Delta \psi_{o}$, where for 13/6 helix, $\Delta \psi_{o}=2\pi \left(6/13\right)$. The same definitions apply on $\rho_{h,k}^{cg_{i}}$ related to position of the grain centers, $cg_{i}$. Furthermore, discontinuous helices contain finite size atoms, or coarse-grained representations of groups of atoms, which are approximated as effective spheres, with radii, $r_{s}^{a_{i}}$ or $r_{s}^{cg_{i}}$.

In cylindrical coordinates, a continuous helix or a helical wire is defined as $r=r_{0}$, ψ=2πz/P, z=z. The Fourier transform in 3D in cylindrical coordinates has the form

(A1) $$\hat{f}\left(R,\Psi ,Z\right)=\frac{1}{{\left(2\pi \right)}^{3/2}}\;\int_{0}^{+\infty }\int_{0}^{2\pi }\int_{-\infty }^{+\infty }f\left(r,\psi ,z\right)\cdot e^{-i2\pi \left[rR\;\cos\left(\Psi -\psi \right)+zZ\right]}\cdot r\cdot drd\psi dz$$

where $f\left(r,\psi ,z\right)$ is the object function, R,Ψ,Z are cylindrical coordinates in inverse space, the exponential term is the scalar product of coordinates in real and inverse space and rdrdψdz is the infinitesimal volume. Using the identity $\cos\left(\Psi -\psi \right)=\sin\left(\Psi -\psi +\pi /2\right)$ for the exponential term in Equation (A1) and expressing the sine function as $\sin\left(\Psi -\psi +\pi /2\right)=\left(e^{i\omega }-e^{-i\omega }\right)/2i=\left(t-t^{-1}\right)/2i$, where ω=Ψ−ψ+π/2 and $t=e^{i\omega }$, the Equation (A1) can be re-expressed using a Bessel function expansion [55] as

(A2) $$\hat{f}\left(R,\Psi ,Z\right)=\frac{1}{{\left(2\pi \right)}^{3/2}}\sum_{n=-\infty }^{+\infty }e^{in\cdot \left(\Psi +\frac{\pi }{2}\right)}\;\int_{0}^{+\infty }\int_{0}^{2\pi }\int_{-\infty }^{+\infty }f\left(r,\psi ,z\right)\cdot J_{n}\left(2\pi rR\right)\cdot e^{-in\psi }\cdot e^{i2\pi zZ}\cdot r\cdot drd\psi dz$$

The transform derived for a continuous helix [15,56] applies to helix starting from ψ=0 when z=0. However, for a rotated identical helix, whether for atom $a_{i}$ or coarse-grained actin monomer substructure, such that $\psi =\psi_{0}$ when $z=z_{o}$, the transform should include an angular exponential term to account for the shift in angle of $\psi_{0}$.

### Appendix A.2. The Fourier Transform of a Continuous Helix

Let us here consider first the Fourier transform for a nonuniformly deformed continuous helix passing through an atom $a_{i}$, with radius, $r_{o}^{a_{i}}$, defined as [15,56] $d\psi /dz=2\pi /P\left(z\right)$, where a piecewise pitch, P(z), is represented as a set of the constant pitches, $P_{l}$, within segments l (Figure 13B). The step changes from $P_{l}$ to $P_{l+1}$ occur at the axial locations where crossbridges are attached, $z_{l}$. The helix object function $f\left(r,\psi ,z\right)$, assuming that the density along the helix is unity, can be expressed for each atom $a_{i}$ as the product of two δ-functions:

(A3) $$f^{a_{i}}\left(r,\psi ,z\right)=\delta \left(r-r_{0}^{a_{i}}\right)\cdot \delta \left(\psi^{a_{i}}-\frac{2\pi z^{a_{i}}}{P\left(z\right)}\right)$$

where $\psi^{a_{i}}$ denotes helix passing through atom $a_{i}$ starting at the angular position $\psi_{o}^{a_{i}}$, P(z) is represented as the series of $P_{l}$ values at each segment $L_{l}=z_{l}-z_{l-1}$ (Figure 13A). The number of these constant pitch segments is $l_{\max}$.

After integration over r and ψ using the formalism $\int \delta \left(x-x_{0}\right)\cdot f\left(x\right)dx=f\left(x_{0}\right)$, the Fourier transform for continuous helix passing through atom $a_{i}$ (Equation (2)) becomes

(A4) $${\hat{f}}^{a_{i}}\left(R,\Psi ,Z\right)=\frac{r_{o}^{a_{i}}}{{\left(2\pi \right)}^{3/2}}\sum_{n=-\infty }^{+\infty }e^{in\cdot \left(\Psi +\frac{\pi }{2}+\psi_{o}^{a_{i}}\right)}\cdot J_{n}\left(2\pi r_{o}^{a_{i}}R\right)\;\int_{-\infty }^{+\infty }e^{i2\pi \left(Z-\frac{2\pi z}{P(z)}\right)z}\cdot dz$$

### Appendix A.3. The Fourier Transform of a Discontinuous Helix

The Fourier transforms of uniformly and nonuniformly deformed discontinuous helices, $\hat{f}\left(R,\Psi ,Z\right)$, were derived in Prodanovic et al. [15]. In their formulation, each actin monomer is lumped into a single equivalent sphere, representing all atoms in the monomer, and the force transferred from the myosin crossbridge to the actin coincides with the axial location of the equivalent sphere center. Because the axial tension, strain, and intermonomer spacing along an actin filament change at each site of bound myosin, i.e., in a single plane perpendicular to the helix axis, the piecewise description of deformed actin filament is directly defined by the axial coordinate $z_{l}$ from MUSICO simulations. The assumption that crossbridge force is transferred to the actin filament within a single plane perpendicular to the helix axis at $z_{l}$, rather than across an interaction area, simplifies mathematical formulation. Since monomer spacing in deformed actin filament varies from segment to segment, and multiple monomers can be within each segment, we included both the current monomer number, m, and the current segment, l, in the notation of axial and angular positions of atom $a_{i}$. Therefore, in the following text, the coordinates in deformed configuration are denoted as $z_{m,l}^{a_{i}}$ and $\psi_{m,l}^{a_{i}}$.

## Appendix B

### Discontinuous Helices Derived from All-Atom Actin Filament Structure Under Relaxed and Contracted Conditions

The axial periodicity of the atoms in the relaxed filaments will follow equidistant spacings of the axial subunits. Each atom forms its own discontinuous helix with a radius $r_{0}^{a_{i}}$ and uniform axial spacing of the subunits $p_{o}$. The positions of the helices are defined by the coordinates of the atom $a_{i}$ in the first monomer $r_{0}^{a_{i}}$, $\psi_{m=1}^{a_{i}}$ and $z_{m=1}^{a_{i}}$, where the axial position of the same atom on all other subunits is assumed to be on a continuous helix of constant radius, starting at the angular position $\psi_{o}^{a_{i}}=\psi_{1}^{a_{i}}-\left(2\pi z_{1}^{a_{i}}\right)/P_{o}$ at $z=z_{o}=0$ (Figure 13B,C). The coordinate $z_{0}$ can be defined arbitrarily, but because of simplicity we define it as the coordinate at the tip of actin filament, i.e., the position of atom which is farthest away from the plane passing through the center of mass of the first monomer. The coordinates of atom $a_{i}$ in monomer m following equidistant spacings between the sequential monomers along actin filaments are $r_{0}^{a_{i}}$, $\psi_{m}^{a_{i}}\;=\;\psi_{1}^{a_{i}}+(m-1)\Delta \psi_{o}$, and $z_{m}^{a_{i}}=z_{1}^{a_{i}}+(m-1)p_{o}$.

In contrast, the axial periodicity of the atoms in the filaments under contracting conditions displays variable spacing between the axial subunits, depending on the tension in segments l between the loading planes. In this configuration, each atom forms its own discontinuous helix with a radius $r_{0}^{a_{i}}$, but axial spacing of the actin monomers aligns with the spacing within each segment l, denoted as $p_{l}^{a_{i}}$, which is proportional to the segment peach, $P_{l}$. This proportionality is valid for all $p_{l}^{a_{i}}$s, except in cases where the spacing spans across two segments, l−1 and l, denoted as intersegment spacings, $p_{l-1,l}^{a_{i}}$. The spacing $p_{l-1,l}^{a_{i}}$ is defined as the difference between the positions of atoms $a_{i}$ in the first monomer of the segment l, $z_{l}^{a_{i},\min}$, and in the last monomer within the segment l−1, $z_{l-1}^{a_{i},\max}$. Since the segments l and segment l−1 contain different pitches, the pitch changes at $z_{l-1}$ from $P_{l-1}$ to $P_{l}$. In deformed configuration fragments, $\Delta z_{l-1}^{a_{i},\max}=z_{l-1}^{a_{i},\max}-z_{l-1}$ and $\Delta z_{l}^{a_{i},\min}=z_{l-1}-z_{l}^{a_{i},\min}$ are derived by assuming that the angular increments between sequential atoms $a_{i}$ remain the same in both deformed and undeformed configurations, $\Delta \psi^{a_{i}}=\Delta \psi_{o}$. This assumption enables calculations of the fragment increments in the deformed configuration by including the changes in pitch, expressed as $\Delta z_{l-1}^{a_{i},\max}=\Delta z_{o}^{a_{i},\max}\left(p_{l-1}/p_{o}\right)$ and $\Delta z_{l}^{a_{i},\min}=\Delta z_{o}^{a_{i},min}\left(p_{l}/p_{o}\right)$, and the intersegment spacing $p_{l-1,l}^{a_{i}}=\Delta z_{l-1}^{a_{i},\max}+\Delta z_{l}^{a_{i},\min}$ (for details see Appendix C).

## Appendix C

Intersegment spacing between successive atoms $a_{i}$ just below and just above load plane l−1, denoted as $p_{l-1,l}^{a_{i}}$, is calculated as the distance between the positions of $a_{i}$ in the last monomer within the segment l−1 and in the first monomer of the segment l (Figure 14). Assuming that the angular increment between consecutive atoms $a_{i}$ on a discrete helix is constant and equal to the angular increment of the undeformed helix, i.e., $\Delta \psi^{a_{i}}=\Delta \psi_{o}$ then the parts of angle increments in the segments l−1 and l are $\Delta \psi_{l-1}^{a_{i},\max}=\psi_{l}^{a_{i}}-\psi_{l-1}^{a_{i},\max}$ and $\Delta \psi_{l}^{a_{i},\min}=\psi_{l}^{a_{i},\min}-\psi_{l}^{a_{i}}$, respectively. Here $\psi_{l}^{a_{i}}$, $\psi_{l}^{a_{i},\min}$ and $\psi_{l-1}^{a_{i},\max}$ are coordinates of the angles of atom $a_{i}$ on the helix passing through the atoms $a_{i}$ in the monomer just below the plane l−1 in segment l−1 and the first monomer above the plane in segment l, respectively. The increment in the intersegment angle is $\Delta \psi_{l-1,l}^{a_{i}}=\Delta \psi_{l-1}^{a_{i},\max}+\Delta \psi_{l}^{a_{i},\min}=\Delta \psi_{o}$.

Accordingly, the parts in intersegment spacing $p_{l-1,l}^{a_{i}}=\Delta z_{l-1}^{a_{i},\max}+\Delta z_{l}^{a_{i},\min}$ can be related to the increments in angle using the definition of helix in deformed configuration as $\Delta \psi_{l-1}^{a_{i},\max}=2\pi \left(\Delta z_{l-1}^{a_{i},\max}/P_{l-1}\right)$ and $\Delta \psi_{l}^{a_{i},\min}=2\pi \left(\Delta z_{l}^{a_{i},\min}/P_{l}\right)$. The sum of the increments in angle, after substituting the above relations, becomes $\Delta \psi_{o}=2\pi \left[\left(\Delta z_{l-1}^{a_{i},\max}/P_{l-1}\right)+\left(\Delta z_{l}^{a_{i},\min}/P_{l}\right)\right]=2\pi \left(6/13\right)$. Since the proportion between monomer spacing and pitch for 13/6 helix in both undeformed and deformed configurations remains the same, if the helix does not unwind, i.e., $p_{l-1}=\left(6/13\right)P_{l-1}$ and $p_{l}=\left(6/13\right)P_{l}$, the above equation simplifies to $\left(\Delta z_{l-1}^{a_{i},\max}/p_{l-1}\right)+\left(\Delta z_{l}^{a_{i},\min}/p_{l}\right)=1$. Furthermore, in the undeformed configuration, the monomer spacing and the intersegment spacing are the same, leading to $\Delta z_{o}^{a_{i},\max}+\Delta z_{o}^{a_{i},\min}=p_{o}$. Because the fragment increments in angles are the same in the deformed and the undeformed configurations, the increments in fragment length in deformed configurations are proportional to the strains in the segments l−1 and l, i.e., $\Delta z_{l-1}^{a_{i},\max}=\Delta z_{o}^{a_{i},\max}\left(p_{l-1}/p_{o}\right)$ and $\Delta z_{l}^{a_{i},\min}=\Delta z_{o}^{a_{i},min}\left(p_{l}/p_{o}\right)$, where the value of $p_{o}$ is defined from the undeformed configuration of the actin filament, and the fragments of atom $a_{i}$ positions relative to the load plane, $\Delta z_{o}^{a_{i},\max}$, and $\Delta z_{o}^{a_{i},\min}$ are known from the undeformed actin filament monomer configuration.

## Appendix D

We have defined five distinct regions where atom $a_{i}$ can be located within the monomer relative to the position on the loading plane l−1. We introduced a differential, denoted as $\Delta \;m_{cs}$, between the monomer number associated with load plane l−1, $m_{l-1}$, and the monomer number where atom $a_{i}$ at coordinate $z_{l-1}^{a_{i},\max}$ is located, $\left(m_{l-1}+\Delta m_{cs}\right)$. This differential is suitable for generalized formulation of the axial coordinate $z_{m,l-1}^{a_{i}}$ for atom $a_{i}$ placed in regions (ii–v) within actin monomer. The value of $\Delta \;m_{cs}$ is equal to zero if $a_{i}$ is in region (ii), 1 if in region (iii), −1 if in region (iv), and −2 if in region (v) (see Figure 14A,B). The monomer number for coordinate $z_{l}^{a_{i},\min}$ is equal to $m_{l-1}+\Delta m_{cs}+1$.

The formulation of the axial coordinate of atom $a_{i}$ in monomer m in the deformed configuration is complex and have to take in account all spacing across the segments, $p_{l}^{a_{i}}$ and intersegment spacings, $p_{l-1,l}^{a_{i}}$, up to the monomer m. When the atom $a_{i}$ is in proximity to the load plane passing through a monomer in a narrow region around the load plane l−1, $z_{l}\pm 0.25$ Å, then $z_{l-1}^{a_{i},\max}\approx \;z_{l}$ or $z_{l}^{a_{i},\min}\approx \;z_{l}$; therefore, $\Delta z_{l-1}^{a_{i},\max}\approx 0$ or $\Delta z_{l}^{a_{i},\min}\approx 0$, thus it is not necessary to calculate $p_{l-1,l}^{a_{i}}$, because $p_{l-1,l}^{a_{i}}\approx p_{l-1}^{a_{i}}$ or $p_{l-1,l}^{a_{i}}\approx p_{l}^{a_{i}}$, respectively. In this case, the number of subunit spacings $p_{l}^{a_{i}}$ within the segment length $L_{l}$ is equal to $M_{l}=L_{l}/p_{l}^{a_{i}}$. This simplifies calculation of $z_{m,l}^{a_{i}}$ in region (i), and coordinate of a monomer m in segment l is simple sum of $p_{l}^{a_{i}}$ s over the segments up to (and including) l−1, and the spacings in segment l up to m:

(A5) $$z_{m,l}^{a_{i}}=z_{1,1}^{a_{i}}+\sum_{ii=1}^{l-1}\left(M_{ii}\right)\cdot p_{ii}+\left[m-\left(1+\sum_{kk=1}^{l-1}M_{kk}\right)\right]p_{l}$$

except in segment l=1 when the coordinate simplifies to $z_{m,l=1}^{a_{i}}$ is $z_{m,1}^{a_{i}}=z_{1,1}^{a_{i}}+\left(m-1\right)\cdot p_{1}$.

Note that $p_{1,1}=p_{o}$, because there is no load in the segment l=1, thus the spacing in this segment is the same as in the relaxed filaments.

In all other cases, $p_{l-1,l}^{a_{i}}$ has a value between $p_{l-1}^{a_{i}}$ and $p_{l}^{a_{i}}$, and the monomer numbers from which $\Delta z_{l-1}^{a_{i},\max}$ and $\Delta z_{l}^{a_{i},\min}$ are calculated depend on the region of the monomer where the atom $a_{i}$ is located. Using values of $\Delta \;m_{cs}$ for the atoms placed in the regions (ii–v), enables formulation of general expression for coordinate of a monomer m in segment l as

(A6) $$z_{m,l}^{a_{i}}=z_{1,1}^{a_{i}}+\left(1+\Delta m_{cs}\right)p_{1}+\sum_{ii=1}^{l-1}\left(M_{ii}-1\right)\cdot p_{ii}+\sum_{jj=2}^{l}p_{jj-1,jj}+\left[m-\left(\left(2+\Delta m_{cs}\right)+\sum_{kk=1}^{l-1}M_{kk}\right)\right]\cdot p_{l}$$

This general formula is valid except in situations when crossbridges are bound to the first monomer (m=1). A brief description of how these exceptions are handled is given in Appendix E.

## Appendix E

When crossbridges are bound to the first and second monomer (m=1 and 2), the general formulation related to segment l=1 should be adjusted in the following ways.

If a load plane passes through monomer 1 (m=1) and atom $a_{i}$ is in proximity of the plane (within ±ε) or is below the loading plane (region (ii)), $z_{m=1,l=1}^{a_{i}}=z_{1,1}^{a_{i}}$, then $\Delta z_{1}^{a_{i},\max}=z_{l=1}-z_{1,1}^{a_{i}}<p_{o}$, where $p_{o}$ is the undeformed helix spacing. However, if $\Delta z_{1}^{a_{i},\max}>p_{o}$, then atom $a_{i}$ is also below the load plane, but located within region (iii) of monomer 2 (m=2), and its $\Delta z_{1*}^{a_{i},\max}=z_{l=1}-z_{l=2,m=1}^{a_{i}}=\Delta z_{1}^{a_{i},\max}-p_{o}$. Note that below the load plane, there is no deformation, thus, in the segment l=1, the spacing remains unchanged (i.e., $p_{1}\equiv p_{o}$). Once $\Delta z_{1}^{a_{i},\max}$ or $\Delta z_{1*}^{a_{i},\max}$ are calculated using Equation (A6), then $p_{1,2}^{a_{i}}$ can be obtained, as described above.

On the other hand, when atom $a_{i}$ position is *above* the load plane, all atoms are in regions (iv and v), then there is no a single atom $a_{i}$ of monomer m=1 in the segment l=1, and the atom $a_{i}$ position of the first monomer is in the segment l=2. In this case, part of the monomer above the loading plane should be corrected for the deformation in the segment l=2, i.e., $z_{m=1,l=2}^{a_{i}}=z_{l=1}+(z_{1,2}^{a_{i}}-z_{l=1})\left(p_{l=2}/p_{o}\right)$. Since the atom $a_{i}$ in m=1 is in segment l=2, there is no intersegment spacing $p_{1,2}^{a_{i}}$. In addition, below the load line there is no deformation of the monomer thus for actin filament end part from $z_{o}$ to $z_{l=1}$ is in undeformed, whereas part of the monomer above the load line is in the deformed part of the filament, so this part of the coordinate $z_{m=1,l=2}^{a_{i}}$ includes correction for the strain $p_{l=2}/p_{o}$ because this part belongs to the segment l=2.

Moreover, when crossbridges are bound to monomer 1 and 2, and also for atoms $a_{i}$ in monomer m=1 which have a position above the (load) plane l=2, i.e., $>z_{l=2}$ for atoms $a_{i}$ belonging to region (v), then there is not a single atom $a_{i}$ of monomer m=1 in the segments l=1 and l=2. Therefore, positions of the atoms $a_{i}$ of the first monomer are in the segment l=3, and their axial coordinate should be corrected for the deformation in the segments l=2 and l=3, i.e., $z_{m=1,l=3}^{a_{i}}=z_{l=1}+(p_{o})\left(p_{l=2}/p_{o}\right)+(z_{1,2}^{a_{i}}-z_{l=1}-p_{o})\left(p_{l=3}/p_{o}\right)$. Note that, in this case, there is no $p_{1,2}^{a_{i}}$ and $p_{2,3}^{a_{i}}$, and the deformation in segments l=2 and l=3 are included in the corrected coordinate $z_{m=1,l=3}^{a_{i}}$. Also, below the load line l=1 there is no deformation of the monomer, thus, $z_{l=1}$ is in undeformed part, but part of the monomer above the load line is in the deformed part of the filament, so this part of the coordinate $z_{m=1,l=3}^{a_{i}}$ includes correction for the strain $p_{l=2}/p_{o}$ because this part belongs to the segment l=2 and also part of the coordinate $z_{m=1,l=3}^{a_{i}}$ includes correction for the strain $p_{l=3}/p_{o}$ because this part belongs to the segment l=3.

Because the coordinate of the atom $a_{i}$ in the first monomer is in segment 2 or in segment 3, the lower and upper limits of summation in the Equation (A6) should be adjusted appropriately.

## Appendix F

The intensity is proportional to ${\left|\hat{f}\left(R,\Psi ,Z\right)\right|}^{2}=\hat{f}\left(R,\Psi ,Z\right)\cdot {\hat{f}}^{*}\left(R,\Psi ,Z\right)$, i.e., the Fourier transform (Equation (1)) should be multiplied by its complex conjugate. Because the intensity, ${\left|\hat{f}\left(R,\Psi ,Z\right)\right|}^{2}$, varies with Ψ, cylindrically averaged diffraction intensity is then obtained by integration of the intensity over Ψ from 0 to 2π. The analytic expression for cylindrically averaged diffraction intensity can then be easily derived for finite number of layer lines as a sum of products of the coefficients $G_{n}\left(R,Z\right)$, and the nonzero values of the corresponding integrals over all layer lines. Thus, the cylindrical average after integration simplifies to

(A7) 12π∫02πf^R,Ψ,Z⋅f^*R,Ψ,Z dΨ=∑n=1∞ReGn2+ImGn2

Detailed derivation of the Equation (A7) is shown in Appendix from Prodanovic [15].

For the numerical integration of Equation (A7), we used the trapezoidal rule by dividing the integrand into equal angular segments, ΔΨ wide, and summing their areas to approximately obtain the cylindrically averaged actin filament X-ray diffraction pattern. Using an angular increment of ΔΨ=2π/18 provided sufficient accuracy. For details see Supplemental Material (Figures S8 and S9 and Tables S1 and S2).

## Appendix G

**Table A1:** Alphabetical list of symbols used throughout the manuscript, with corresponding definitions.

Symbols	Definition
$a_{i}$	Individual atom with index i
$cg_{i}$	Individual coarse-grained sphere with index i
$f\left(r,\psi ,z\right)$	Helix object function
${\hat{f}}^{a_{i}}\left(R,\Psi ,Z\right)$	The Fourier transform for a discontinuous helix passing through atom $a_{i}$
$f_{l}$	Crossbridge force acting on actin
$G_{n}\left(R,Z\right)$	Fourier transform of helical structure factor $g_{n}\left(r,Z\right)$
$G_{s}^{a_{i}}$	Fourier transform of spherical object, for specific atom $a_{i}$
$J_{n}$	Bessel function of the n-th order
l	Current number of the plane where myosin is bound to actin
$l_{\max}$	Total number of constant pitch segments
$L_{l}$	Axial length of segment l with constant pitch $P_{l}$
m	Current number of a subunit (monomer)
$m_{l}$	Monomer indexed l
$m_{l-1}$	Monomer indexed l−1
$\Delta \;m_{cs}$	The differential used for generalized formulation of the axial coordinate $z_{m,l-1}^{a_{i}}$ for atom $a_{i}$ placed in regions (ii–v) within actin monomer; determines monomer index shift.
$M_{l}$	Number of monomer spacings within segment $L_{l}$
$N_{a_{i}}$	Number of atoms in the actin monomer
p	Axial spacing between monomers along the helix
$p_{o}$	Axial spacing between monomers along the helix for the undeformed helix.
$p_{l}$	Axial spacing between monomers along the helix within segment l
$p_{l-1,l}^{a_{i}}$	Intersegment monomer spacing of atom $a_{i}$ between successive monomers, depending of the position of atom $a_{i}$ within the monomer and $\Delta z_{l-1}^{a_{i},\max}$ and $\Delta z_{l}^{a_{i},\min}$, and associated strains on each side of the load plane l−1
$P_{o}$	Pitch of the undeformed helix.
$P_{l}$	Pitch within the segment l
P(z)	Piecewise pitch represented as a set of the constant pitches, $P_{l}$, within segments l
ψ	Helical angular coordinate
Δψ	Azimuthal angle increment
$\psi_{l}$	Azimuthal angular position of the helix passing through the load plane
$\psi_{m,l}^{a_{i}}$	Azimuthal angle of atom $a_{i}$ in monomer m within segment l
$\psi_{o}^{a_{i}\left(or\;cg_{i}\right)}$	Starting angular coordinate of discontinuous helix for each atom and for each grain
$\Delta \psi_{o}^{a_{i}\left(or\;cg_{i}\right)}$	Angular increment between atoms $a_{i}$ in sequential monomers
$\psi_{o}^{cc_{i}}$	Angular position for each cluster center i
$\psi_{l}^{a_{i},\min}$	Angular coordinates of the angles of atom $a_{i}$ on the helix passing through the atoms $a_{i}$ in the monomer just below the plane l−1, in segment l−1
$\psi_{l-1}^{a_{i},\max}$	Angular coordinates of the angles of atom $a_{i}$ on the helix passing through the atoms $a_{i}$ in the first monomer above the plane in segment l
$r_{o}$	Helix cylinder radius for discontinuous helix
$r_{0}^{a_{i}}$	Helix radius of an atom in the undeformed helix configuration
$r^{a_{i}}$	Helix radius for each atom $a_{i}$
$r_{m}^{a_{i}}$	Radial coordinate of atom $a_{i}$ in monomer m.
$r_{o}^{cc_{i}}$	Radial distance from the actin filament’s main axis for each cluster center i
$r_{s}$	Equivalent radius used to present grain size
$r_{s}^{a_{i}}$, $r_{s}^{cg_{i}}$	Effective sphere radius for groups of atoms and coarse grained representations
$r_{vdw}^{a_{i}}$	Van der Waals radius of atom $a_{i}$
rdrdψdz	Infinitesimal volume
R,Ψ,Z	Cylindrical coordinates in inverse space
$\rho_{h}$	Continuous helical wire with pitch P
$\rho_{k}$	Density planes separated by axial distance p
$\rho_{h,k}$	Discontinuous helix, which can be constructed as the product of a continuous helical wire, $\rho_{h}$, with a pitch, P, and a set of horizontal density planes, $\rho_{k}$
$\rho_{h,k}^{a_{i}}$, $\rho_{h,k}^{cg_{i}}$	Discontinuous helix cylinder radius for each atom and for each grain
$\rho_{s}^{a_{i}}$	Transform of finite-size atom object
$V_{eff}^{a_{i}}$	Effective atomic volume
z	Helix axial coordinate
$z_{0}$	Axial coordinate at the tip of actin filament (starting axial position of the actin filament)
$z_{o}^{cc_{i}}$	Axial position for each cluster center i
$\Delta z_{o}^{a_{i},\max}$,$\Delta z_{o}^{a_{i},\min}$	Fragments of atom $a_{i}$ positions relative to the load plane
$\Delta z_{1}^{a_{i},\max}$	Increment of axial coordinate for atom $a_{i}$ in monomer 1 relative to the loading plane
$\Delta z_{1*}^{a_{i},\max}$	Increment of axial coordinate when atom is in region (iii) of monomer 2 but below the load plane
$z_{l}$	Midplane through the interaction area where a crossbridge is bound, corresponds to plane l
$z_{l_{\max}}$	Coordinate of the last plane
$z_{l-1}^{a_{i},\max}$	Maximum coordinate of atom $a_{i}$ relative to the load plane in monomer l−1
$z_{l}^{a_{i},\min}$	Minimum coordinate of atom $a_{i}$ relative to the load plane in monomer l
$z_{m}^{a_{i}}$	Axial coordinate of atom $a_{i}$ in monomer m, measured along the filament axis in undeformed configuration
$z_{m,l}^{a_{i}}$	Axial position of atom $a_{i}$ in subunit m within segment l in nonuniformly deformed configuration
