# Supplementary material for: Comparison of Predicted X-Ray Fiber Diffraction Patterns from All-Atom and Coarse-Grained Actin Filament Models Under Nonuniform Strain
Source: Int J Mol Sci. 2025 Dec 26;27(1):280. doi: 10.3390/ijms27010280 (PMC12786265; doi:10.3390/ijms27010280)
Supplement: Supplementary file 1 [file ijms-27-00280-s001.zip › ijms-3992617-supplementary.pdf]

Supplementary Material

1. Representing the Actin Monomer with Grains of the Equivalent Sizes

The X-ray diffraction pattern of a discontinuous helix of finite size atoms, a_i , is calculated as the product of the Fourier transforms of the discontinuous helix of finite length, $\rho_k^{a_i}$, and of the atom size, $\rho_s^{a_i}$. The atom size and electron density are obtained for each atom as its Van der Waals radius, $r_{vdw}^{a_i}$ from the chosen actin atomic structure (*dom4b.pdb*). For coarse-grained structures, the same approach is used, where the equivalent sphere radius is calculated from the volume of all atoms in the coarse-grained structure. The actin monomer atomic structure partitioned into a predetermined number of clusters (e.g., 1, 9, 47, 260, and 1016 clusters) using the k-means clustering algorithm. The grain volume is then computed as the cumulative volume of the atoms contained within each cluster. Because the number atoms and distribution of atom sizes vary from clusters to cluster, their sizes vary and in the calculation of X-ray diffraction patterns actual grain sizes were used. For illustration, in Fig. S1 are shown: 3D distribution of the atom centers within an actin monomer (A), and then in (B) are, in addition, included atomic size spheres. In Figs. S1 C, D and F are shown actin monomer representations by single equivalent sphere, nine spheres and 47 spheres. The spatial coordinates of grain centers and the grain equivalent volumes sizes, obtained from our clustering algorithm calculations, are used for the predictions of X-ray Diffraction patterns.

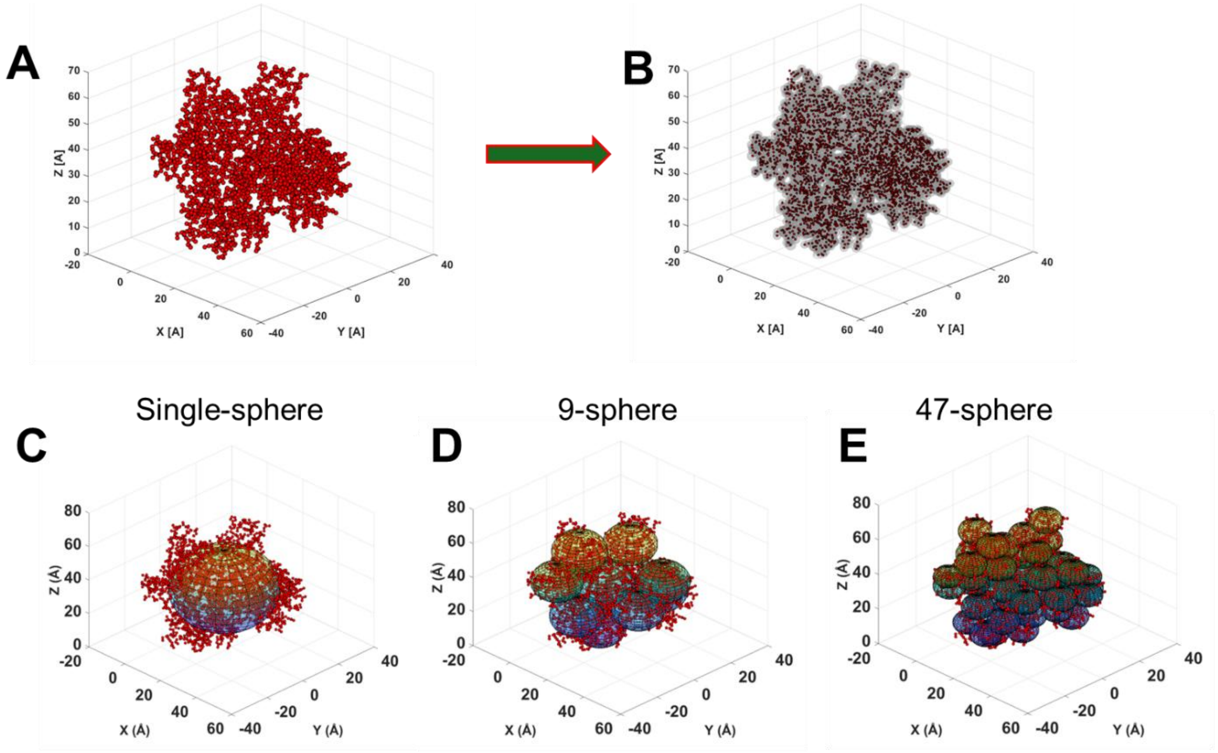

Figure S1. Coarse-grained representations of an actin monomer. (A) Spatial distribution of atomic centers extracted from the actin monomer structure (*dom4b.pdb*). (B) The complete actin monomer is illustrated with effective atomic volumes, highlighting the spatial distribution of electron density. Coarse-grained models of the actin monomer generated using our clustering procedure are presented in panels (C)–(E): (C) a single-sphere representation, (D) a 9-sphere model, and (E) a 47-sphere model.

2. Sensitivity analysis of Predicted X-ray Diffraction Patterns on Structural Model Parameters of Actin Filaments

Diffraction features in predicted X-ray diffraction patterns are strongly affected by the discontinuous helix cylinder radius r_o, and the grain size represented as an equivalent sphere radius, r_s^{eq} , which, in the limiting case, reduces to atomic dimensions. To understand their individual contributions, we performed a sensitivity analysis focusing on these parameters. Specifically, we explored the effects of the helix cylinder radius r_o, passing through the center of an atom or a coarse-grained structure, as well as the effect of atomic size or equivalent grain size on the predicted X-ray diffraction pattern.

2.1 The Effects of Helix Cylinder Radius on the Predicted X-ray Diffraction Pattern

The helix cylinder radius, r_o, affects the magnitude of reflections and the shape of the Bessel functions. We conducted sensitivity analyses using three different helix radii: the radius corresponding to the center of mass of the actin monomer, $r_o^{a_i}$ =16.2 Å, a smaller radius of 10 Å, and a larger radius of 30 Å. The predicted intensities at these three radii, as well as the differential intensities between predicted patterns at $r_o^{a_i}$ =16.2 Å and $r_o^{a_i}$ =10 Å, and also between $r_o^{a_i}$ =16.2 Å and $r_o^{a_i}$ =30 Å, are shown in Fig. S2.

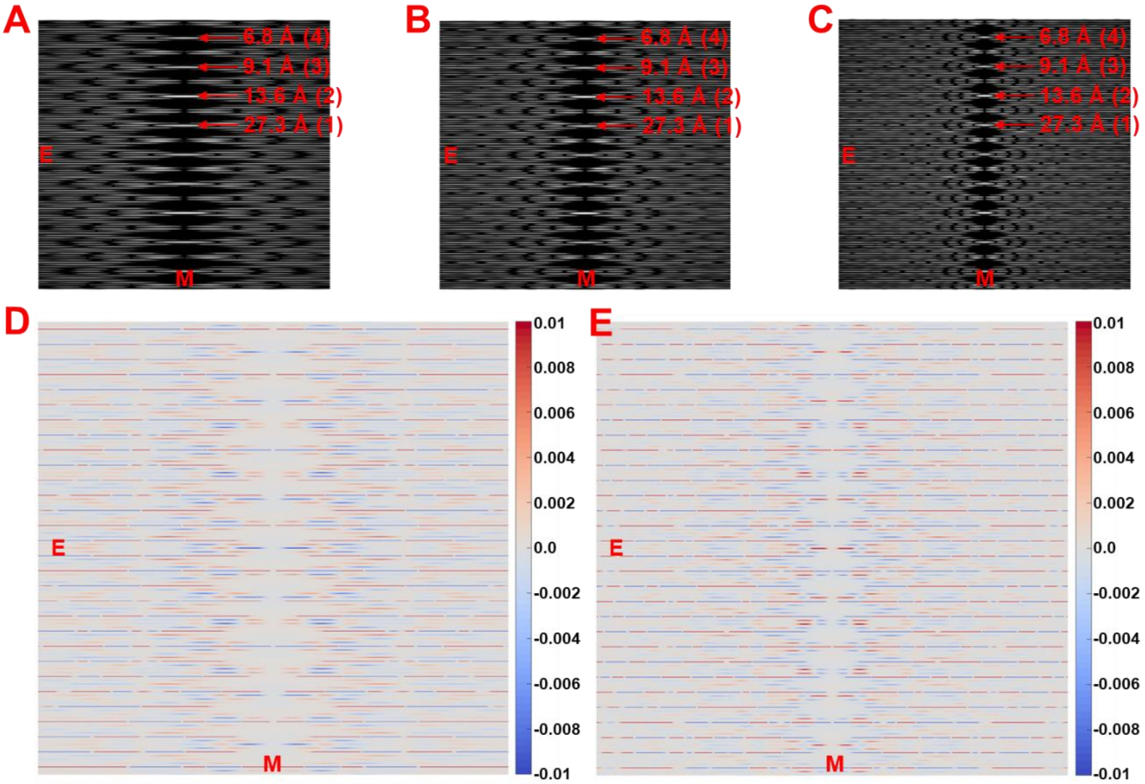

Figure S2. Sensitivity analysis of discontinuous helix radius on predicted X-ray diffraction intensities. Three helix radii were examined: $r_o^{a_i}$ = 10 Å (A), smaller than the discrete helix that passes through center of the actin monomers ($r_o^{a_i}$ = 16.24 Å) (B) and larger radius at $r_o^{a_i}$ = 30 Å (C). The differences in normalized intensities of X-ray diffraction patterns by $r_o^{a_i}$ between discrete helices at $r_o^{a_i}$ = 10 Å vs. 16.24 Å (D), and images (D) and (E) only minimal differences are observed near the meridional axis, but the differences significantly and progressively increase along the layer lines in the radial direction.

Notably, the meridional reflections remain unaffected by variations in the helix cylinder radius, displaying no significant shifts in their positions or changes in their shape. This observation supports previous analysis by (Prodanovic *et al.*, 2016, Mijailovich *et al.*, 2019), confirming that using a single discontinuous helix, passing through the centers of mass of the actin monomers is sufficient for interpreting spacing changes of the meridional reflections observed during muscle contractions.

While variations due to the helix cylinder radius are subtle in predicted 2D diffraction patterns (Fig. S2, panels A–C), their impact is more clearly revealed through difference maps. Fig. S2D shows the differences between 2D patterns at the helix radii of 16.2 Å and 10 Å, while Fig. S2E illustrates the differences between 16.2 Å and 30 Å. These difference maps demonstrate that variations in the helix cylinder radius significantly affect radial profiles along the layer lines, which must be accounted for in diffraction modeling.

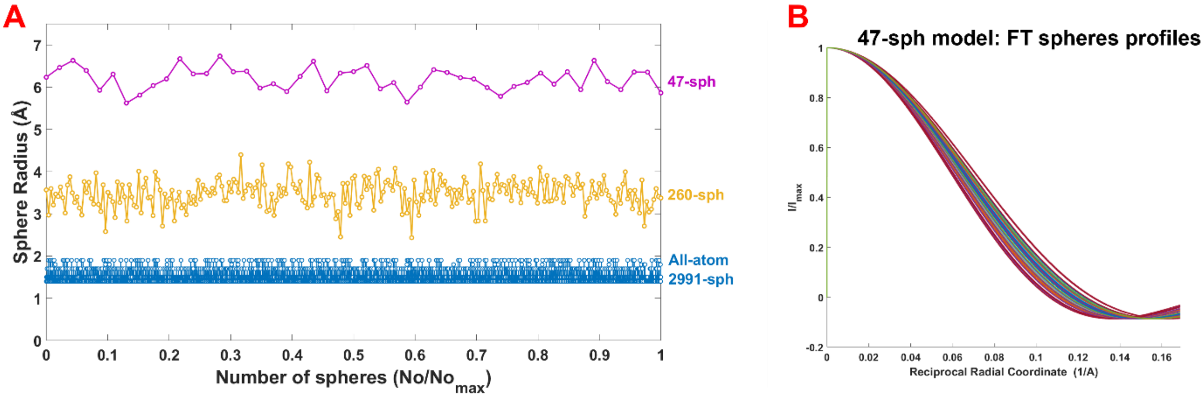

Figure S3. A) Variability of sphere radii for each CG model normalized to maximum number of spheres. B) Profiles of Fourier transforms of spheres with varying radii for 47-sphere model.

2.2 The Effects of Equivalent Grain Size on the Predicted X-ray Diffraction Pattern

The equivalent grain size, defined by radius of equivalent sphere, r_s , limits the number of visible layer lines (Fig. S4), *i.e.* the axial extent of the X-ray diffraction pattern, and as well as the observable range in the radial direction. In all-atom simulations, the predicted X-ray diffraction patterns extend far beyond the fourth actin meridional reflection. However, as the grain size increases to r_s = 6.2 Å, only the first two actin meridional reflections remain visible, and the observable pattern rapidly decreases with further increase in grain size. At r_s = 10.75 Å, only the

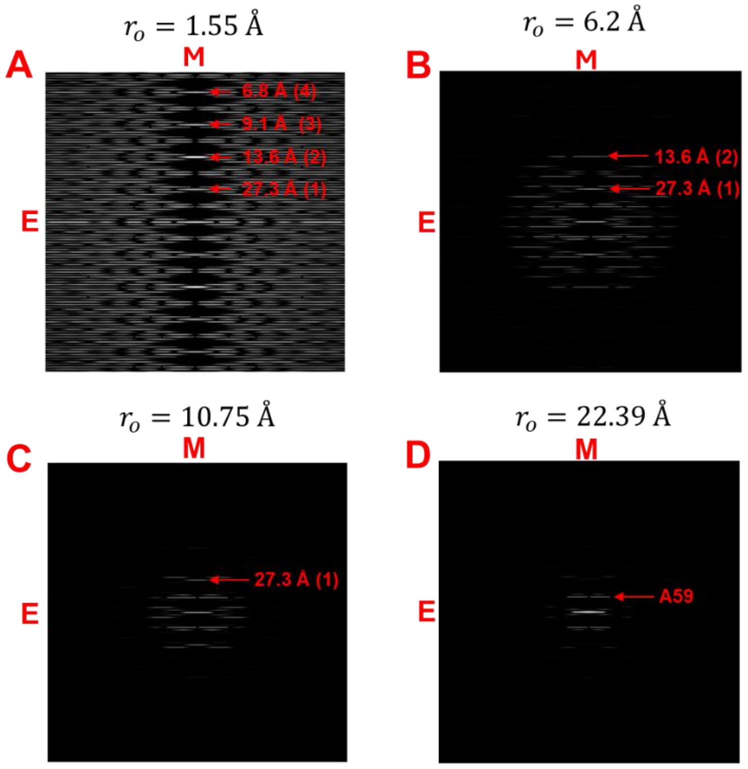

Figure S4. Sensitivity analysis of grain size on X-ray diffraction patterns of actin monomers represented by coarse-grained model. The diffraction patterns for the single-sphere model at helix radius cylinder r_o = 16.24 Å are compared for different equivalent sphere radii (r_s): 1.55 Å (atomic-scale), 6.2 Å, 10.75 Å, and 22.39 Å. The largest radius represents the equivalent sphere corresponding to the total mass of the actin monomer. As r_s increases, the number of visible actin meridional reflections decreases: more than four reflections are clearly visible at r_s =1.55 Å, up to two reflections at 6.2 Å, up to one at 10.75 Å, while the 6th layer line remains visible for the full monomer at 22.39 Å. All intensities are scaled by a specified linear factor.

first actin meridional reflection is observable. Similarly, the visibility of features in the radial direction is significantly diminished with increasing the radius of the grain. All intensities are scaled by a specified linear factor.

The rapid decay of intensity along the layer lines in the radial direction with increasing equivalent radius, r_s^{eq}, is evident in the comparison of equatorial profiles (Fig. S5). Multiple peaks observed for atomic-size spheres (r_s^{eq} = 1.55 Å) are significantly reduced at r_s^{eq} = 6.2 Å, and nearly disappear at r_s^{eq} > 15 Å. A similar decrease in intensity with increasing r_s^{eq} is also observed in the meridional reflections (Fig. S6). The decrease in the second meridional reflection (Fig.S6 B) limits its visibility to coarse-grained models with 47 spheres or more. Higher-order actin meridional reflections, *e.g.* 3rd and 4th, require much finer resolution, with models composed of at least 260 spheres.

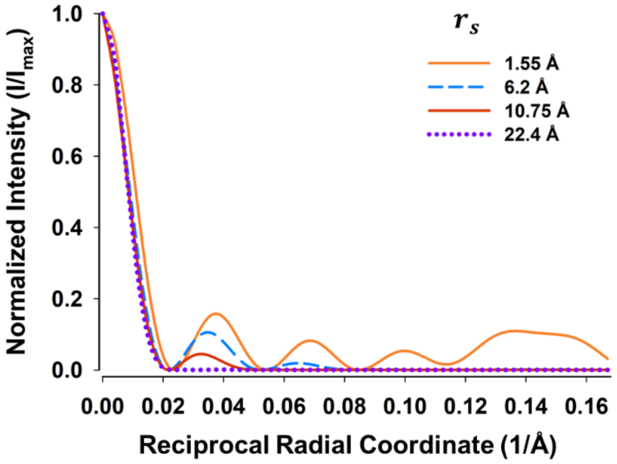

Figure S5. Sensitivity analysis of actin monomers represented by coarse-grained model on X-ray diffraction equatorial profiles. The equatorial profiles are compared between sphere radii 1.55 Å (orange solid line), 6.2 Å (blue dashed line), 10.75 Å (red solid line), and 22.39 Å (purple dotted line). The profiles demonstrate variations in peak amplitudes, while the positions of the peaks are preserved. Finer-grained models exhibit more pronounced peaks at lower scattering angles. Helix cylinder radius of 16.24 Å is used in all simulations.

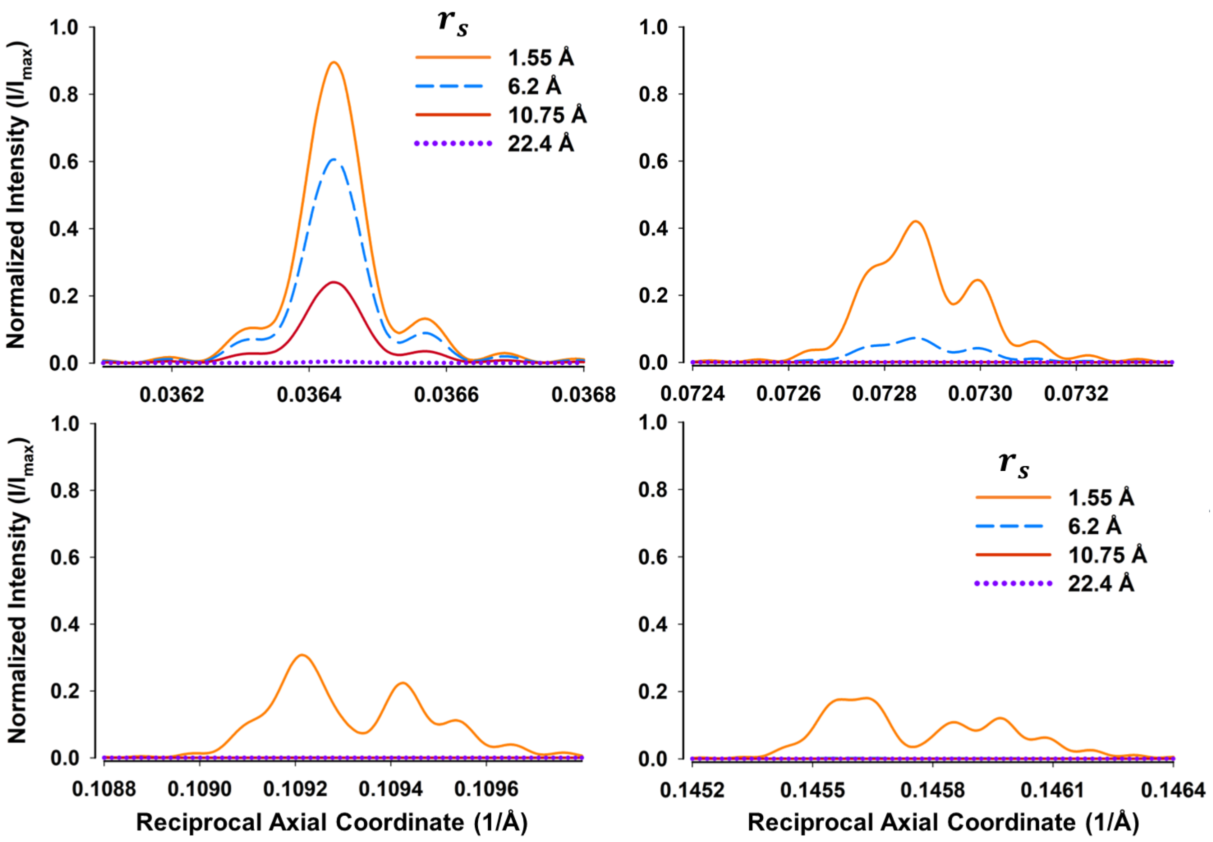

Figure S6. Sensitivity Analysis of actin meridional reflections profiles with respect to grain size (equivalent sphere radius, r_s^{eq}). The meridional profiles are compared between sphere radii of 1.55 Å (red solid line), 6.2 Å (blue dashed line), 10.75 Å (green solid line), and 22.39 Å (purple dotted line). The first reflection is visible for all grain sizes, although it is barely detectable at 22.39 Å (A). The second reflection is present only for the 1.55 Å and 6.2 Å models (B), while the third (C) and fourth (D) reflections are observed exclusively for the 1.55 Å model. This behavior is consistent with the shape of the Fourier transform of a sphere, where the first zero limits the spatial frequency content retained by the model (see Table 1). Models with smaller equivalent sphere radii preserve higher-resolution features and more closely approximate all-atom predictions.

2.3. X-ray Diffraction Patterns Predicted from Coarse-Grained Structural Model of Actin Filaments with Eqcuivalent Grain Size Spheres.

The equivalent grain size, defined by the radius of equivalent sphere, r_s, used to approximate the structure of the actin monomer multi-atom substructure limits the number of visible layer lines (Fig. 8), *i.e.* the axial extent of the X-ray diffraction pattern, and as well as the range of reciprocal spacings in the radial direction where features will be visible. The intensity distributions in the patterns will be modulated by the intensity transform (form factor) of the spheres constituting the coarse-grains with the radius of their first zero values in the transform given in Table 1 and indicated by a red dotted circle in the images which matches circular regions of low intensity in the images (Fig. 8). Predicted X-ray diffraction patterns in all-atom simulations yield diffraction features far beyond the fourth actin meridional reflection but as the grain size increases to $r_s = 6.2$ Å (47 spheres per actin monomer), only the first three actin meridional reflections are visible and rapidly decreases with increasing grain size, so that when $r_s = 10.75$ Å (9 spheres per actin monomer) only the first actin meridional reflection is seen.

The effect of grain size on the prediction of X-ray diffraction patterns is illustrated in Fig. S7. The predictions of X-ray diffraction patterns of an all-atom discrete helix system, compared with those with either 47 and 260 spheres per actin monomer for both relaxed and contracted muscle, showed significant differences in the intensity distributions in the 2D patterns (Fig. S7 A, B, C), most obviously between the 47-sphere and all-atom models. The effect of the distribution in

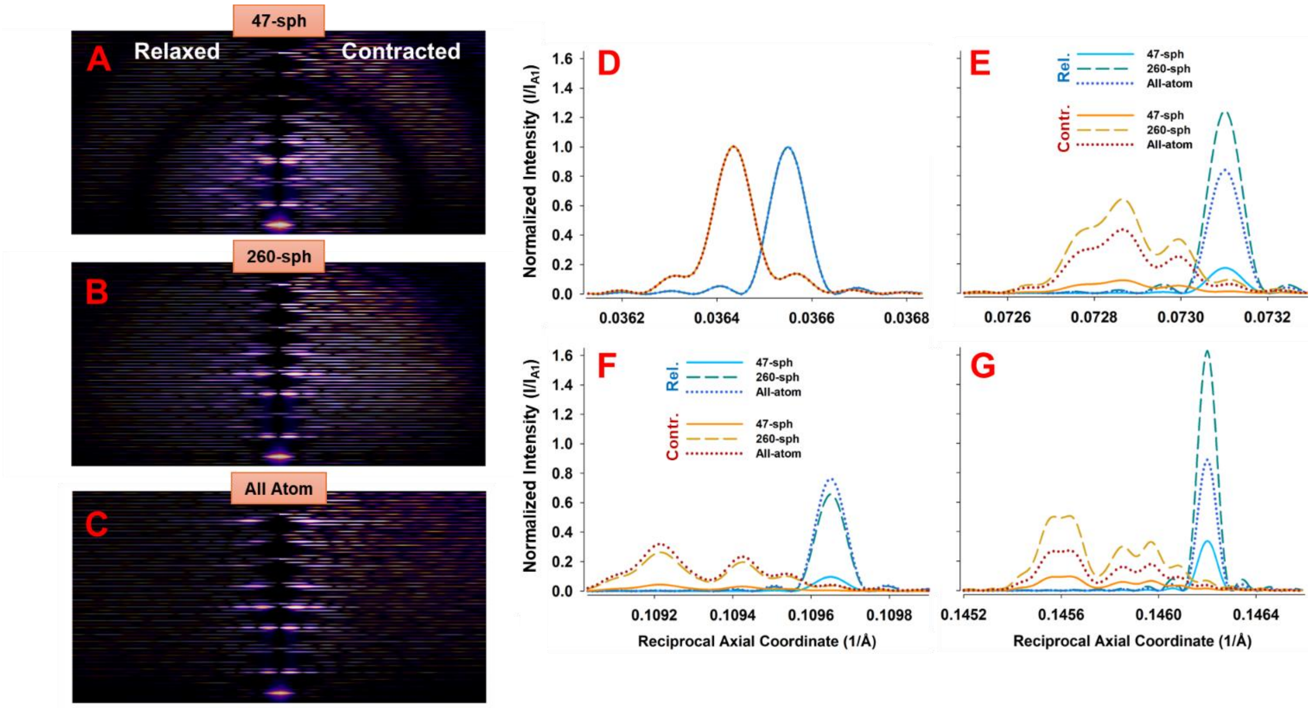

Figure S7. Comparison of X-ray diffraction patterns and meridional intensity profiles between relaxed and contracted actin filaments using models with different levels of coarse-graining: 47-sphere model (A), 260-sphere model (B), and all-atom model (C). Intensity profiles of the 1st to 4th actin meridional reflections are shown in panels D–G, respectively. Blue lines represent relaxed filaments, while orange lines correspond to contracting filaments. The 47-sphere coarse-grained model is shown as solid lines, the 260-sphere model as dashed lines, and the all-atom model as dotted lines. The overall peak positions remain similar across different model resolutions, but the intensity varies with the level of coarse-graining. As the number of spheres in the model increases, the diffraction intensities become closer to those of the all-atom model.

monomer spacings in the deformed configuration on meridional reflections had minimal effect on the first actin meridional reflection (Fig. S7 D). whereas the second, third and fourth meridional reflections (Figs. S7 E, F, G respectively), were strongly influenced by grain size r_s . Note that the contracting (deformed) profiles were reasonably close in shape and intensity profiles from the 260 sphere and all atom models for the second and third meridional reflections with less good agreement on the 4th meridional reflection while retaining a similar overall shape.

2.4 Numerical convergence of the trapezoidal azimuthal averaging scheme

To assess the numerical convergence of the trapezoidal azimuthal averaging scheme ($\Delta\gamma = 2\pi/18$), we computed X-ray patterns using 3, 6, 9, and 18 azimuthal sampling angles for contracted filaments modelled at three levels of structural detail: all-atom, a 47-sphere coarse-grained representation, and a single-sphere model in which each monomer is replaced by a sphere of atomic radius (~1.55 Å) positioned at the monomer's center of mass. All intensities were normalized to the number of azimuthal samples. For the meridional reflections (1st–4th orders), all sampling schemes produced identical intensities within numerical precision; differences were at the level of numerical noise ($<10^{-10}$ %), indicating complete convergence even when a single azimuthal angle is used. For the 6th layer line, small fluctuations were detectable between sampling schemes, but they remained within noise levels and had no meaningful effect on the radial profile.

More noticeable differences appeared on layer lines containing significant radial features. Along the 14th layer line (which intersects the first actin meridional reflection), deviations between the 6-angle and 18-angle samplings were <0.03%, and those between the 9-angle and 18-angle samplings were <0.01% for the all-atom model. Slightly larger but still very small differences were observed for the 47-sphere and single-sphere models (≤0.56% for 6 angles and ≤0.12% for 9 angles). Only the 3-angle sampling produced visibly distinct profiles, reaching deviations of ~2% at higher reciprocal spacings (from 1/27.4 Å to 1/6.25 Å), with the largest differences occurring in the all-atom model and decreasing systematically in the coarse-grained models. A comparable pattern was seen on the 28th layer line (intersecting the second actin meridional reflection): deviations remained <1% for both 6- and 9-angle samplings across all models, but rose sharply for the 3-angle scheme, reaching values as high as 13% at the highest spatial frequencies. Complete numerical results and convergence figures are provided in Figures S8 and S9 and Tables S1 and S2.

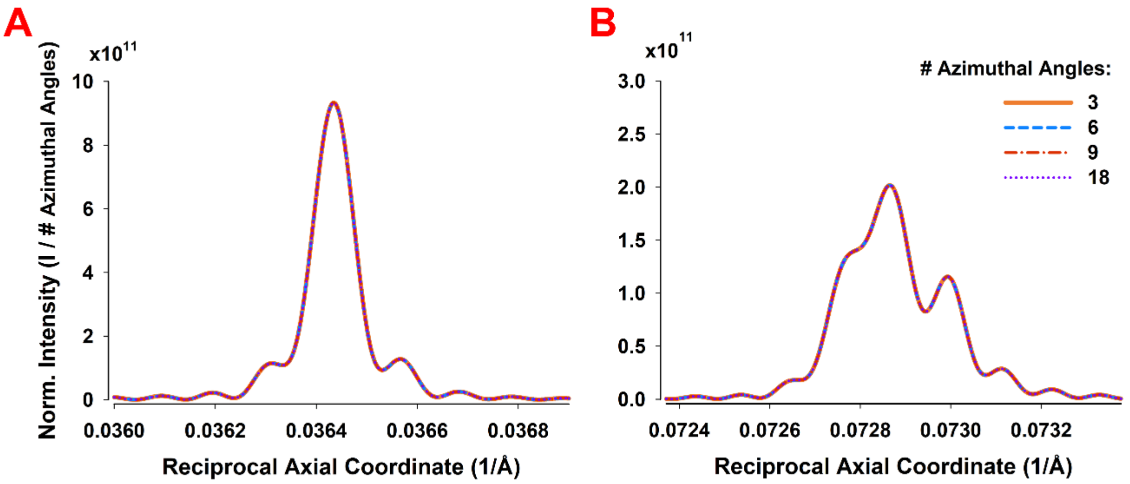

Figure S8. Axial intensity profiles of the first (A) and second (B) actin meridional reflections computed using 3 (orange solid lines), 6 (blue dashed lines), 9 (red dash-dotted lines), and 18 (violet dotted lines) azimuthal angles and normalized by the number of samples. The identical peak positions and intensities demonstrate full convergence and insensitivity of meridional reflections to azimuthal averaging.

Overall, the analysis indicates that the meridional reflections are completely insensitive to azimuthal averaging and can be computed accurately even with a single azimuthal angle. For the radial profiles, a single angle remains adequate up to resolutions of ~1/13.6 Å, with only minimal differences appearing beyond this point. At higher spatial frequencies the profile shape becomes more sensitive to the number of azimuthal samples, and averaging over six angles is already sufficient to reproduce the 18-angle reference with high accuracy. Using nine angles produces results that are essentially indistinguishable from the 18-angle calculation.

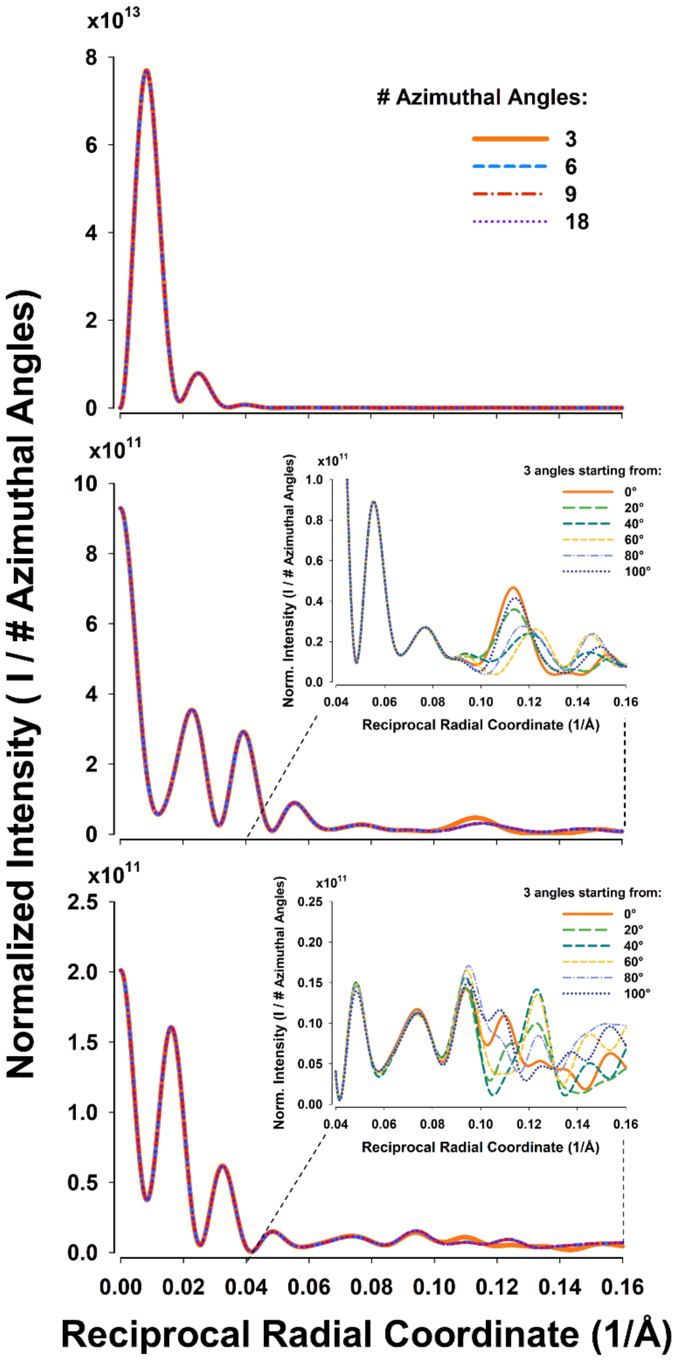

Figure S9. Radial intensity profiles of the 6th (top panel), 14th (middle panel), and 28th (bottom panel) layer lines computed using 3 (orange solid lines), 6 (blue dashed lines), 9 (red dash-dotted lines), and 18 (violet dotted lines) azimuthal angles and normalized by the number of samples. The 14th and 28th layer lines intersect the first and second actin meridional reflections, respectively; insets in panels B and C highlight the sensitivity of the profiles to the starting angle when only three azimuthal samples are used.

Table S1. Percentage differences in integrated intensity along the 14th layer line relative to the 18-angle azimuthal averaging reference for the all-atom, 47-sphere, and single-sphere models. The 1/d ranges 0.00–0.04 Å⁻¹ and 0.04–0.16 Å⁻¹ correspond approximately to d ≥ 27.4 Å and 6.25 Å ≤ d ≤ 27.4 Å, respectively.

14th layer line					
			**All-atom**	**47-sphere**	**Single sphere**
**Angles**	**Initial azimuth (°)**	**1/d range (Å⁻¹)**	**% difference vs 18 angles**	**% difference vs 18 angles**	**% difference vs 18 angles**
3	0	0.00–0.04	-0.006	0.044	-0.048
3	20	0.00–0.04	0.047	-0.233	0.034
3	40	0.00–0.04	0.011	0.065	0.054
3	60	0.00–0.04	-0.003	0.059	0.034
3	80	0.00–0.04	0.008	-0.274	-0.006
3	100	0.00–0.04	-0.057	0.338	-0.068
3	0	0.04–0.16	1.694	0.424	-0.736
3	20	0.04–0.16	-0.202	1.056	0.194
3	40	0.04–0.16	-1.967	-0.150	1.077
3	60	0.04–0.16	-1.703	-1.544	0.073
3	80	0.04–0.16	0.238	-0.624	-0.109
3	100	0.04–0.16	1.940	0.838	-0.499
6	0	0.00–0.04	-0.005	0.052	-0.007
6	20	0.00–0.04	0.027	-0.254	0.014
6	40	0.00–0.04	-0.023	0.202	-0.007
6	0	0.04–0.16	-0.005	-0.560	-0.331
6	20	0.04–0.16	0.018	0.216	0.042
6	40	0.04–0.16	-0.014	0.344	0.289
9	0	0.00–0.04	0.004	-0.055	0.000
9	20	0.00–0.04	-0.004	0.055	0.000
9	0	0.04–0.16	-0.012	-0.117	0.077
9	20	0.04–0.16	0.012	0.117	-0.077
18	–	0.00–0.04	0.000	0.000	0.000
18	–	0.04–0.16	0.000	0.000	0.000

Table S2. Percentage differences in integrated intensity along the 28th layer line relative to the 18-angle azimuthal averaging reference for the all-atom, 47-sphere, and single-sphere models. The 1/d ranges 0.00–0.04 Å⁻¹ and 0.04–0.16 Å⁻¹ correspond approximately to d ≥ 27.4 Å and 6.25 Å ≤ d ≤ 27.4 Å, respectively.

		28th layer line			
			All-atom	47-sphere	Single sphere
Angles	Initial azimuth (°)	1/d range (Å⁻¹)	% difference vs 18 angles	% difference vs 18 angles	% difference vs 18 angles
3	0	0.00–0.04	-0.089	-0.147	-0.027
3	20	0.00–0.04	-0.171	-0.317	0.096
3	40	0.00–0.04	-0.021	0.061	0.091
3	60	0.00–0.04	-0.139	0.124	-0.022
3	80	0.00–0.04	0.168	0.068	-0.066
3	100	0.00–0.04	0.253	0.211	-0.072
3	0	0.04–0.16	-6.649	5.804	-1.113
3	20	0.04–0.16	-12.827	-4.231	2.041
3	40	0.04–0.16	-4.855	-9.392	-0.354
3	60	0.04–0.16	7.801	-4.247	0.141
3	80	0.04–0.16	13.174	3.744	1.002
3	100	0.04–0.16	3.356	8.323	-1.718
6	0	0.00–0.04	-0.114	-0.011	-0.025
6	20	0.00–0.04	-0.002	-0.125	0.015
6	40	0.00–0.04	0.116	0.136	0.009
6	0	0.04–0.16	0.576	0.778	-0.486
6	20	0.04–0.16	0.173	-0.244	1.522
6	40	0.04–0.16	-0.750	-0.535	-1.036
9	0	0.00–0.04	0.019	-0.006	0.000
9	20	0.00–0.04	-0.019	0.006	0.000
9	0	0.04–0.16	0.557	0.052	-0.155
9	20	0.04–0.16	-0.557	-0.052	0.155
18	–	0.00–0.04	0.000	0.000	0.000
18	–	0.04–0.16	0.000	0.000	0.000

References

Mijailovich, S. M., Prodanovic, M. & Irving, T. C. (2019). *Int J Mol Sci* **20**.

Prodanovic, M., Irving, T. C. & Mijailovich, S. M. (2016). *J Appl Crystallogr* **49**, 784-797.
